# Supplementary material for: Effectiveness of protected areas in conserving tropical forest birds
Source: Nat Commun. 2020 Sep 14;11:4461. doi: 10.1038/s41467-020-18230-0 (PMC7490714; doi:10.1038/s41467-020-18230-0)
Supplement: Supplementary file 1 — Supplementary Information [file 41467_2020_18230_MOESM1_ESM.pdf]

This file includes Supplementary Information to the article "Effectiveness of protected areas in conserving tropical forest birds" by Cazalis et al.

Contents

Supplementary Methods **3**
1. Data selection: eBird checklists . . . 3
   A. Spatial filtering . . . 3
   B. Filtering by sampling protocol . . . 3
   C. Observation filtering . . . 4
   D. Filtering checklists based on observer experience . . . 4
   E. Removing duplicates . . . 5
   F. Taxonomic standardisation . . . 5
   G. Final dataset analysed . . . 6
2. Site characteristics . . . 6
   A. Protection (binary) . . . 6
   B. Forest habitat (binary) . . . 6
   C. Altitude (continuous) . . . 7
   D. Agricultural suitability (continuous) . . . 7
   E. Remoteness (continuous) . . . 7
   F. Canopy height (continuous; forest sites only) . . . 7
   G. Forest contiguity (continuous; forest sites only) . . . 7
   H. Wilderness level (continuous; forest sites only) . . . 7
   I. Deforestation rates (continuous; all sites) . . . 7
   J. Overall species richness . . . 8
   K. Richness in forest-dependent species . . . 8
   L. Richness in endemic species . . . 8
   M. Richness in threatened and Near Threatened species . . . 8
3. Index of observer expertise . . . 9
4. Statistical analyses of protected area effectiveness . . . 9
   A. Analysis I: effect of protected areas on bird diversity . . . 10
   B. Analysis II: effect of protected areas on forest quantity and quality 11
   C. Analysis III: effect of forest presence and quality on bird diversity . 12
   D. Potential effects of differences in habitat between protected and non-protected sites . . . 13
   E. Potential effect of protected area age on effectiveness . . . 15

Supplementary Tables **16**

Supplementary Figures **19**

Supplementary Discussion **37**

References **38**

Supplementary Methods

1. Data selection: eBird checklists

A. Spatial filtering

We focused on global biodiversity hotspots[1] which overlapped by more than 25% of their extent the "tropical and subtropical moist broadleaf forests" biome (boundaries from Olson et al.[2]). We obtained 16 hotspots: Atlantic Forest, Tropical Andes, Tumbes-Chocó-Magdalena, Caribbean Islands, Mesoamerica, Guinean Forests of West Africa, Eastern Afromontane, Coastal Forests of Eastern Africa, Madagascar, Western Ghats and Sri Lanka, Indo-Burma, Sundaland, Philippines, East Melanesian Islands, and New Caledonia.

Of these, we ultimately analysed only eight hotspots for which we obtained more than 1,000 eBird checklists (after following the data filtering procedure detailed below). We first downloaded the eBird checklists for the respective countries per hotspot (eBird codes between brackets):

- **Atlantic Forest:** Argentina [AR], Brazil [BR], Paraguay [PY].
- **Tropical Andes:** Argentina [AR], Bolivia [BO], Colombia [CO], Ec-uador [EC], Peru [PE], Venezuela [VE].
- **Tumbes-Choco-Magdalena:** Colombia [CO], Ecuador [EC], Panama [PA], Peru [PE].
- **Mesoamerica:** Belize [BZ], Costa Rica [CR], Guatemala [GT], Hon-duras [HN], Mexico [MX], Nicaragua [NI], Panama [PA], El Salvador [SV].
- **Eastern Afromontane:** Burundi [BI], Democratic Republic of Congo [CD], Eritrea [ER], Ethiopia [ET], Kenya [KE], Mozambique [MZ], Rwanda [RW], Sudan [SD], South Sudan [SS], Tanzania [TZ], Uganda [UG].
- **Western Ghats and Sri Lanka:** India [IN], Sri Lanka [LK].
- **Indo-Burma:** Bangladesh [BD], Hong Kong [HK], India [IN], Cam-bodia [KH], Laos [LA], Myanmar [MM], Malaysia [MY], Thailand [TH], Vietnam [VN], Chi-na [CN].
- **Sundaland:** Brunei [BN], Indonesia [ID], Malaysia [MY], Thailand [TW].

We then filtered the checklists to include only those overlapping both the hotspot and the "tropical and subtropical moist broadleaf forests" biome.

In the Indo-Burma hotspot, we excluded records from China [CN] (19% of the hotspot, 2% of checklists) because no protected area data were available for this country in the publicly available version of the World Database on Protected Areas[3].

B. Filtering by sampling protocol

In order to be able to treat this dataset as presence/absence, we focused on checklists for which observers stated that they reported every species detected[4]. Accordingly, we

also removed checklists unlikely to capture all species because using particular protocols (e.g. banding) or targeting specific groups (e.g. waders, nocturnal). We therefore used only checklists for which the protocol reported was either 'stationary points' or 'travelling counts'. In stationary points, observers remain at the checklist location, and report both the starting time and the duration of the sampling. In travelling counts, moving observers report the checklist location (usually the mid-point of their itinerary), starting time, duration of the sampling and distance travelled. We excluded travelling counts with a travel distance > 5km, as they may not represent the local bird composition around the reported GPS location. To further increase comparability, we excluded sampling events that were very short (< 30minutes) or very long (> 10hours).

We also included some data classified in the eBird dataset under the protocol category 'historical counts', which consist of sampling events for which birding was the primary focus but for which the observer was not able to fill all fields required for reporting stationary points or travel counts (e.g. starting time, duration, distance). We used historical counts if duration was known and ranged from 30 minutes to 10 hours, and if distance was known and shorter than 5 km.

C. Observation filtering

We excluded observations that were disapproved by the eBird review process, corresponding to exotic, feral or escaped individuals. Established introduced species were kept in the dataset.

Using the *auk rollup* function from the above-mentioned R package 'auk'[5], we brought all observations of subspecies to the species level.

D. Filtering checklists based on observer experience

To aim for complete checklists, we filtered observations to retain only those submitted by relatively experienced observers. In order to identify these, we analysed checklists per observer across each of three continents, defined according to the following list of countries (eBird codes between brackets):

- **Americas:** Antigua and Barbuda [AG], Anguilla [AI], Argentina [AR], Boliv-ia [BO], Brazil [BR], Bahamas [BS], Belize [BZ], Chile [CL], Colombia [CO], Costa Rica [CR], Cuba [CU], Dominica [DM], Dominican Republic [DO], Ecuador [EC] Falkland Islands [FK], Grenada [GD], French Guyana [GF], Guadeloupe [GP], Guatemala [GT], Guyana [GY], Honduras [HN], Haiti [HT], Jamaica [JM], Saint Knitts and Nevis [KN], Cayman Is-lands [KY], Saint Lucia [LC], Martinique [MQ], Montserrat [MS], Mexico [MX], Nicaragua [NI], Panama [PA], Peru [PE], Puerto Rico [PR], Paraguay [PY], Suriname [SR], El Savador [SV], Turks and Caicos Islands [TC], Uruguay [UY], Saint Vincent and the Grenadines [VC], Venezuela [VE], British Virgin Islands [VG], Virgin Islands [VI].

- **Asia:** Bangladesh [BD], Brunei [BN], Bhutan [BT], China [CN], Hong Kong [HK], Indonesia [ID], India [IN], Cambodia [KH], Laos [LA], Sri Lanka [LK], Myanmar [MM], Malaysia [MY], Nepal [NP], Papua New Guinea [PG], Philippines [PH], Pakistan [PK], Thailand [TH], Taiwan [TW], Vietnam [VN].

- **Africa:** Angola [AO], Burkina Faso [BF], Burundi [BI], Benin [BJ], Botswa-na [BW], Democratic Republic of Congo [CD], Central African Republic [CF], Congo [CG], Cote d'Ivoire [CI], Cameroon [CM], Djibouti [DJ], Algeria [DZ], Egypt [EG], Western Saha-ra [EH], Eritrea [ER], Ethiopia [ET], Gabon [GA], Ghana [GH], Gambia [GM], Guinea [GN], Equatorial Guinea [GQ], Guinea-Bissau [GW], Kenya [KE], Liberia [LR], Lesotho [LS], Lib-ya [LY], Morocco [MA], Madagascar [MG], Mali [ML], Mauritania [MR], Malawi [MW], Mozambique [MZ], Namibia [NA], Niger [NE], Nigeria [NG], Rwanda [RW], Sudan [SD], Sierra Leone [SL], Senegal [SN], Somalia [SO], South Sudan [SS], Sao Tomé and Principe [ST], Swaziland [SZ], Chad [TD], Togo [TG], Tunisia [TN], Tanzania [TZ], Uganda [UG], South Africa [ZA], Zambia [ZM], Zimbabwe [ZW].

Within each given continent, we defined as 'experienced observers' those who had submitted $\geq$ 10 checklists to eBird, with $\geq$ 30 species per checklist on average, and covering $\geq$ 100 different species in total. We only retained the checklists by the observers who were classified as 'experienced' in the corresponding continent.

E. Removing duplicates

Multiple observations of the same birds can happen either because several observers travelled together or because they came independently to the same site on the same day, both situations creating pseudo-replication.

When submitting checklists, observers can specify if they were observing with others. For checklists in this situation, we removed duplication by merging checklists, using the *auk unique* function implemented in the R package 'auk' (a package specifically created to process eBird data[5]) and keeping the number of observers as covariates for all analyses.

In addition, we filtered for other possible duplicates by independent observers: whenever two checklists with equal dates were reported with less than 2 km between them, we randomly selected one of them.

F. Taxonomic standardisation

The taxonomic classification used in eBird follows the Clements taxonomy[6]. In order to be able to cross the bird observation dataset with the species' trait data (section 2 below) we have converted it to the taxonomy used by BirdLife International and HBW[7]. For this, we used an unpublished table kindly provided by the Cornell Lab of Ornithology, which summarises relationships between the two taxonomies, by applying the following rules:

- In the case of a simple difference in name, we applied the Birdlife name to the eBird records (295 species).
- Whenever a single species in the BirdLife list was treated as multiple species in eBird, we lumped the eBird records (92 species).
- Whenever multiple species in the BirdLife list were treated as a single species in eBird, we split the eBird records based on the BirdLife distribution maps for the corresponding species (358 species). Any records outside the BirdLife distribution maps were assigned to the species whose distribution was the closest. In the extreme

rare case of overlap between distributions of these species (12 over the 5,467 species for a total of $\sim 1,500$ observations), observations falling within the distribution overlap were all assigned to a single of the two species (selected randomly between both).

The lists of species in each case are detailed in the Supplementary Data 1.

G. Final dataset analysed

After the above steps, plus the removal of sites of intermediate forest cover (see section 2A below), we obtained a total of 66,777 checklists, covering 5,467 species, from 6,838 observers, in eight hotspots. The list of species per hotspot is available in Supplementary Data 2. This was the final dataset used in the analyses. For further details, and a breakdown per hotspot, see Supplementary Table 2 and Supplementary Figures 4-5.

2. Site characteristics

Our analyses include two types of sites: checklist sites, corresponding to the coordinates of each eBird checklist analysed (used in analyses I and III, see below); and background sites, corresponding to the centre points of a regular grid of 2*2 km covering evenly the whole area of each hotspot (used in analysis II).

We characterised each site according to five variables: two binary (protected vs. non-protected; forest vs. non-forest) and three continuous (altitude; agricultural suitability; remoteness). For forest sites, we characterised them according to three additional continuous variables (canopy height; forest contiguity; and wilderness level).

Checklist sites were also characterised according to four measures of local bird diversity (richness of all species, of forest-dependent species, of endemic species, and of threatened and Near Threatened species).

A. Protection (binary)

A site was considered 'protected' if its coordinates overlapped a protected area, as mapped in the World Database on Protected Areas[3]. As is the standard protocol in global analysis of protected area coverage[8], we excluded: "Man and Biosphere" reserves; protected areas without associated polygons; protected areas that did not have as status "designated", "inscribed" or "established".

We would have obtained similar results if we had instead derived the protection status from the proportion of area under protection within a 1-km buffer around the site, as the vast majority of buffers are protected by either 0 or 100% (Supplementary Figure 6).

B. Forest habitat (binary)

To derive whether a site was forested or not, we used the 2015 version of Climate Change Initiative Land Cover layer, with a resolution of 300m[9]. We considered as forests all categories described as strict Tree Cover (i.e. codes 50-90, 160 and 170), and as non-forest all others (but excluding from the analyses water bodies, code 210).

For each site, we first calculated the percentage of pixels overlapping the 1-km buffer that were forest. We then classified as 'forest' the sites with $> 60\%$ forest, and as 'non-forest' those with $< 10\%$ forest. We thus obtained two types of localities very contrasting

in their forest cover, removing from the analyses all sites with intermediate (10 to 60%) cover.

C. Altitude (continuous)

Altitude data were obtained from the GLOBE Digital Elevation Model[10], which has a 0.008 degree resolution ($\sim 930m$ at latitude 0). We calculated the altitude per site as the median of the values intersecting a 1-km buffer around the site.

D. Agricultural suitability (continuous)

We used a global raster of resolution $\sim$ 1-km mapping a value of agricultural suitability[11]. Their model estimates for each cell the suitability of each of the 16 most important food and energy crops in the world (based on climatic conditions, soil and topography) and assigns to the cell the value of the crop with the highest suitability. It has no unit and is included in a 0 - 100 interval. We obtained a value of agricultural suitability per site as the median of the values intersecting a 1-km buffer around the site.

E. Remoteness (continuous)

Remoteness was derived from the global accessibility map of resolution $\sim$ 1-km, which estimates the travel time needed for a human to reach the nearest city with $\geq 1,500$ inhabitants[12]. We obtained a remoteness value per site as the median of the values intersecting a 1-km buffer around the site.

F. Canopy height (continuous; forest sites only)

We used a global raster of canopy height at resolution $\geq$ 1-km, limited to maximum canopy heights of 40m, derived from spaceborne light detection and ranging (lidar) data[13]. We calculated the canopy height value for all forest sites as the median of the values intersecting a 1-km buffer around the site.

G. Forest contiguity (continuous; forest sites only)

Using the above-mentioned forest layer (used to classify sites as forest or not), we assigned to each forest site the proportion of forest cover (0.6 to 1) as an index of forest contiguity.

H. Wilderness level (continuous; forest sites only)

We used the 2009 global terrestrial human footprint map[14], with a resolution $\sim$ 1-km, obtained by combining spatial information on human pressures including human infrastructures, agricultural land use and population density. For each forest site, we obtained a wilderness value as the opposite of the median human footprint (-1 * human footprint) across pixels intersecting the 1-km buffer around the site.

I. Deforestation rates (continuous; all sites)

We used a global map of forest loss between 2000 and 2019 based on Landsat imagery, which has a resolution of $\sim 30m$[15]. For each background buffer (regardless of the

Non-forest/Intermediate/Forest classification), we calculated the proportion of pixels that experienced forest loss between 2000 and 2019.

J. Overall species richness

For each checklist site, we calculated the total number of species detected in the checklist.

K. Richness in forest-dependent species

For each checklist site, we calculated the total number of species classified as 'forest-dependent'. These are species classified as either highly- or medium-dependent in a pre-existing classification by BirdLifeInternational[16] that includes five categories:

- **Highly-dependent:** Forest specialists; characteristic of the interior of undisturbed forest; may persist in secondary forest and forest patches if their particular ecological requirements are met, but where they do occur away from the interior, they are usually less common; rarely seen in non-forest habitats; breeding is almost invariably within forest.
- **Medium-dependent:** Forest generalists; may occur in undisturbed forest but also regularly found in forest strips, edges and gaps; likely to be commoner in such situations and in secondary forest than in the interior of intact forest; breeding is typically within forest.
- **Low-dependent:** Often recorded in forest, but not dependent on it; almost always more common in non-forest habitats, where most likely to breed.
- **Non-forest species:** Does not normally occur in forest.
- **Unknown (none in the dataset):** Occurs or probably occurs in forest, but dependency on it is unknown, but could be high.

L. Richness in endemic species

For each checklist site, we calculated the total number of species that are classified as 'endemic' to the corresponding hotspot. This includes all species with at least 90% of its global distribution[7] contained within the boundary of the hotspot (considering the whole area of the hotspot, not only the part included in the "tropical and subtropical moist broadleaf forests" biome).

M. Richness in threatened and Near Threatened species

For each checklist site, we calculated the total number of species classified as being either threatened (Vulnerable, Endangered, or Critically Endangered) or Near Threatened in the International Union for Conservation of Nature (IUCN) Red List of Threatened Species[16].

3. Index of observer expertise

For each observer in our analysis, we derived an expertise score using an index adapted from Kelling et al.[17] and from Johnston et al.[18]. Calculated separately for each continent, the index estimates the variation in the number of species that observers are predicted to detect in similar conditions.

We first ran a mixed General Additive Model (function *gamm* from 'mgcv' R package[19]) modelling the species richness of checklists against several sampling variables that are expected to affect species richness, adding observer (i.e., observer individual identifying number) as a random effect:

$gamm(richness \sim protocol + n.observers + s(duration) + s(time) + te(lon, lat, day) + random = list(observer \sim 1))$ (1)

To control for differences in checklist sampling effort, we included both *duration* (of sampling, in minutes) and *time* (starting hour of sampling) as smooth terms, allowing non-linear correlations. We included *lon* (longitude, in decimal degrees), *lat* (latitude, in decimal degrees) and *day* (Julian date, from 1 to 365/366) as a smoothed three-way interaction, thus allowing richness to vary across space and season of the year. We opted for a GAM (following Kelling et al.[17]) rather than a Generalised Linear Model (as Johnston et al.[18]) because the former allows for non-linear effects. For fitting issues, we only included a random effect on intercept, rather than also on the slope between duration and richness, as in Kelling et al.[17].

Following Johnston et al.[18], we fitted this model to a nearly complete dataset per continent (defined as in section 1D), i.e., before the filtering steps detailed in sections 1 and 2A. The only filtering rules applied were to exclude: observations prior to 2005; disapproved observations; checklists that did not report all species observed (because the model is based on richness). We also removed checklists that did not report one of the covariates (about 15% of observations), given that the GAMM cannot accommodate empty records.

We assumed that species richness followed a Poisson distribution, as in Kelling et al.[17] and Johnston et al.[18], because the dataset includes many checklists with low richness, making the distribution closer to a Poisson than a Gaussian distribution.

Having fitted the model to the data, we then used it to predict the species richness that each observer would report for a fictive stationary point with all variables fixed to their median values. The observer expertise score, measured as the logarithm of this predicted species richness, ranged from 2.2 to 4.3 in Africa, from 2.3 to 4.4 in the Americas, and from 2.8 to 4.5 in Asia. We then assigned to each checklist used in our analysis the expertise score of the observer. In case of multiple observers, we assigned the score of the observer with the highest expertise score. This score was then used as an explanatory variable in the statistical analyses below.

4. Statistical analyses of protected area effectiveness

We investigated protected area effectiveness at retaining bird diversity through a set of three connected statistical analyses (Fig. 2). These analyses were undertaken separately for each hotspot to allow for variations across hotspots in the relations between bird di-

versity and covariates (e.g., altitude, remoteness, protection), and because sampling effort is too heterogeneous between hotspots. We used General Additive Models (GAMs) for all analyses, which are similar to Generalised Linear Models, but accommodate nonlinear relationships between response and explanatory variables[19, 20].

We implemented these models using the 'mgcv' R package[19], running an independent model for each hotspot and for each response variable.

In analyses I and III, we assumed a negative binomial distribution for all models, except for those where overall species richness was the response variable. For the later, the distribution obtained after the filtering of checklists was closer to a Gaussian distribution, so we assumed that instead. In analysis II, we assumed a Binomial distribution for forest presence and Gaussian distributions for the three variables of forest quality.

A. Analysis I: effect of protected areas on bird diversity

Analysis I quantifies the effect of protected areas on the bird diversity reported in checklists, controlling for site location biases and other potential confounding factors. The model has the following structure:

$Bird\_Diversity \sim protection + location\_biases + control$ (2)

where:

- $Bird\_Diversity$ corresponds to one of the four bird diversity indices: overall richness; richness in forest-dependent species; richness in endemics; richness in threatened and Near Threatened species;
- $Protection$ corresponds to the binary variable indicating whether the site is protected (1) or not (0);
- $location\_biases$ corresponds to a term controlling for eventual spatial biases on the location of protected areas, formalised as: $s(altitude) + s(remoteness) + s(agricultural\_suitability)$, corresponding to the use of these three variables in independent smoothed terms (allowing non-linear relationships) without limiting the curves complexity (Supplementary Figure 8);
- $control$ corresponds to a term accounting for heterogeneity in sampling effort and potential spatiotemporal variation in bird diversity metrics. In models using the overall species richness as response variable, this was formalised as: $s(duration, k = 4) + s(expertise, k = 4) + s(year, k = 4) + te(day, lat, lon)$. For all other models, we also controlled for overall species richness, so it became: $log(overall\_richness) + s(duration, k = 4) + s(expertise, k = 4) + s(n.observers, k = 4) + s(year, k = 4) + te(lat, lon, day)$. Where:
  - $s(duration, k = 4)$ is the sampling duration in minutes, used here as an independent smoothed term with the degree of the smoothing function fixed to 4, in order to limit the curve complexity (Supplementary Figures 10-17). Results were robust to changes in the degree of smoothing function;
  - $s(expertise, k = 4)$ is the observer expertise score for the checklist, used here as an independent smoothed term with the degree of the smoothing function fixed to 4, in order to limit the curve complexity (Supplementary Figures 10-17);
  - $s(n.observers, k = 4)$ is the number of observers present during the sampling

– $s(year, k = 4)$ is the year of the observation, included to account for potential temporal trends in the region, used here as an independent smoothed term with the degree of the smoothing function fixed to 4, in order to limit the curve complexity (Supplementary Figures 10-17);

– $te(lat, lon, day)$ are the site's decimal coordinates and the Julian date of the observation (from 1 to 365/366), used as a three-way interaction smoothed-term, allowing bird diversity indices to vary spatially during the year (e.g. a species can occur in a region more often during the winter while occurring more often in another region during the summer), thus enabling to account for migration patterns (Supplementary Figures 10-17);

– $log(overall\_richness)$ is the logarithm of the overall species richness. It was used in all models with richness in forest-dependent species, endemic species, and threatened and Near Threatened species as response variable (used in log because we assumed Negative Binomial distributions for these three variables). Therefore, these models test the effect of protection or habitat on the richness in forest-dependent species, endemic species or threatened and Near Threatened species, for a given overall species richness (Supplementary Figures 10-17).

B. Analysis II: effect of protected areas on forest quantity and quality

Analysis II quantifies the effect of protected areas at mitigating forest loss (analysis IIa and IIa') or forest degradation (analysis IIb), controlling for site location biases. Model were built from all background sites (excluding Intermediate forests) within each hotspot. The model structure for analysis IIa is:

$Forest\_presence \sim protection + location\_biases + te(lon, lat)$ (3)

where:

- $Forest\_presence$ corresponds to the binary variable indicating whether the site is forested (1) or not (0);
- $Protection$ corresponds to the binary variable indicating whether the site is protected (1) or not (0);
- $Location\_biases$ corresponds to a term controlling for the possibility of location biases in protected areas (as above) (Supplementary Figure 9);
- $te(lon, lat)$ corresponds to a two-way interaction smoothed term, used here to control for spatial autocorrelation in habitat variables.

The model used for analysis IIa' was identical in its explanatory variables but had as response variable the proportion of forest lost between 2000 and 2019. Models were built from all background sites (including Intermediate forests) with the following structure:

$log(Deforestation\_rates + 0.001) \sim protection + location\_biases + te(lon, lat)$ (4)

where:

- $Deforestation\_rates$ corresponds to the proportion of forest lost between 2000 and 2019. This proportion was log-transformed $[log(Deforestation\_rates + 0.001)]$ so that it fit with a Gaussian distribution;

- $Protection$ corresponds to the binary variable indicating whether the site is protected (1) or not (0);
- $location\_biases$ corresponds to a term controlling for the possibility of location biases in protected areas (as above) (Supplementary Figure 9);
- $te(lon, lat)$ corresponds to a two-way interaction smoothed term, used here to control for spatial autocorrelation in habitat variables.

The models for analysis IIb were restricted to forest sites and have the following structure:

$Forest\_quality \sim protection + location\_biases + te(lon, lat)$ (5)

where:

- $Forest\_quality$ corresponds to one of the three continuous variable used for forest quality: canopy height, forest contiguity, and wilderness;
- $Protection$ corresponds to the binary variable indicating whether the site is protected (1) or not (0);
- $location\_biases$ corresponds to a term controlling for the possibility of location biases in protected areas (as above) (Supplementary Figure 9);
- $te(lon, lat)$ corresponds to a two-way interaction smoothed term, used here to control for spatial autocorrelation in habitat variables.

C. Analysis III: effect of forest presence and quality on bird diversity

Analysis III quantifies the effects of forest presence (IIIa) and forest quality (IIIb) on the bird diversity reported in checklists. The model structure for analysis IIIa is:

$Bird\_Diversity \sim Forest\_presence + control$ (6)

where:

- $Bird\_Diversity$ corresponds to one of the four bird diversity indices: overall richness; richness in forest-dependent species; richness in endemics; richness in threatened and Near Threatened species;
- $control$ corresponds to a term accounting for heterogeneity in sampling effort and potential spatiotemporal variation in bird diversity metrics. It corresponds to the term used in analysis I, but supplemented with $s(altitude, k = 6)$, the altitude in meters, used here as an independent smoothed term with the degree of the smoothing function fixed to 4, in order to limit the curve complexity;

The models for analysis IIIb were restricted to forest sites and have the following structure:

$Bird\_Diversity \sim scale(canopy) + scale(contiguity) + scale(wilderness) + protection + control$ (7)

where:

- $Bird\_Diversity$ corresponds to one of the four bird diversity indices: overall richness; richness in forest-dependent species; richness in endemics; richness in threatened and Near Threatened species;

- *canopy*, *contiguity*, and *wilderness* respectively the canopy height, forest contiguity, and wilderness of checklist sites;
- *scale*() indicates that the variable has been scaled (by subtracting the mean and dividing by the standard deviation), so that their effect size are comparable;
- *Protection* corresponds to the binary variable indicating whether the site is protected (1) or not (0);
- *control* corresponds to a term accounting for heterogeneity in sampling effort and potential spatiotemporal variation in bird diversity metrics. It corresponds to the term used in analysis I, but supplemented with $s(altitude, k = 6)$, the altitude in meters, used here as an independent smoothed term with the degree of the smoothing function fixed to 4, in order to limit the curve complexity;

D. Potential effects of differences in habitat between protected and non-protected sites

In analyses I and IIa, we contrasted protected versus unprotected sites in order to investigate the effects of protection on either bird diversity or on the presence and quality of forest. For this to be a perfect counterfactual analysis, the contrasts ought to have controlled for any confounding effects that make protected sites distinct on average from unprotected ones, in ways besides protection. We considered three types of biases: intrinsic differences in habitat type (e.g. protected sites more likely to be in areas that are naturally forested); differences in deforestation pressure (e.g., if protected areas tend to be in locations with lower likelihood of deforestation, either prior to protection or subsequently); and differences in bird diversity (e.g., if protected areas tend to be located in sites of higher bird diversity). We attempted to reduce these biases by controlling for factors affecting both the initial habitat and subsequent pressures.

Controlling for intrinsic differences in habitat Observed differences in forest cover and bird community composition when contrasting protected versus unprotected sites analysis I and IIa (Figs. 3, 4, 5) could reflect a bias in the location of protected areas towards regions that are naturally forested.

To reduce this bias, we focused analyses within the “tropical and subtropical moist broadleaf forests” biome, which “contains the maximum extent of the world’s tropical and subtropical moist broadleaf forests”[21]. In other words, we tried to ensure that both protected and non-protected areas were naturally forested.

Nonetheless, it is unlikely that this biome would have been 100% forested, as assumed in the analysis. To investigate limits to this assumption, we analysed other maps of historical habitat cover for our study area. A global map of potential vegetation (Ramankutty and Foley[22]; raster at a resolution of 5”, $\sim 9km$ at the Equator), representing the “vegetation that would most likely exist now in the absence of human activities”, predicts that for five out of eight hotspots the area we considered in these analyses would have been originally covered by forests by >80%: Mesoamerica 87%; Tumbes-Chocó-Magdalena 92%; Western Ghats and Sri Lanka 83%; Indo-Burma 90%; Sundaland 96% (considering as forest in the Ramankutty and Foley map: “Tropical Evergreen Woodland”, “Tropical Deciduous Woodland”, “Temperate Evergreen Woodland”, “Temperate Deciduous Woodland”, “Mixed Woodland”). For three other hotspots, it estimates lower proportions of

original forest: Atlantic Forest 69%, Tropical Andes 67%, Eastern Afromontane 30%. Ramankutty and Foley[22]'s method is however likely to underestimate original forest cover by discounting old forest conversion. For the Atlantic Forest, we also analysed a different map of predicted original vegetation produced by the Brazil Institute of Geography and Statistics, according to which our study area was 90% originally covered forest[23]. We were unable to find another reconstruction of original habitat for the whole of the Eastern Afromontane region, but a study focusing on the Eastern Arc Mountain (south-east part of the hotspot) mapped the original forest using paleoecological data and found that it was mostly covered by forest[24]. In the Tropical Andes, much of the disagreement with Ramankutty and Foley[22] corresponds to high altitude grasslands (*páramo*) that were indeed probably not forested (but we also control for altitude, see below). In summary, then, we have high confidence that our study areas were largely (even if not completely) dominated by forest. The differences that remain should be controlled for by our control for altitude in all models, or by our control for spatial autocorrelation.

Controlling for differences in the likelihood of forest loss and degradation Even if sites had similar intrinsic habitats, there may still be differences in habitats between protected and unprotected sites caused by factors other than protection status. In particular, protected areas may be located in regions with lower human pressure (e.g., areas of higher altitude, with less agriculture interest). This in turn may result in differences at the time of creation (protected areas tend to be in the remaining patches of reasonably intact habitat) as well as a progressive separation between protected and unprotected sites, as the latter are more exposed to human pressures. We accounted for this possible bias by controlling in our models (analyses I and IIa) for altitude, agricultural suitability, and remoteness, variables often used in analyses measuring protected area effects on habitat loss[25, 26, 27].

Controlling for potential differences in bird distributions Differences in species richness (overall as well as for particular subgroups of birds) may reflect intrinsic differences in protected versus unprotected sites in their bird composition, for example if protected areas tend to focus on regions that naturally have high bird diversity. We consider this location bias unlikely, as numerous studies have highlighted that protected area location is not usually driven by species distribution but by protection costs[28, 29, 30]. Indeed, these studies found that protected areas have not been demarcated to include sites with higher rates of endemism or threatened species, but areas of low agricultural suitability or potential for other human exploitation, independent from species diversity, which is controlled for in our analyses.

Furthermore, the relatively small scale of our study (using hotspots as units) reduces the variability of bird diversity indices. In addition, some of the covariates used to control for differences in habitat and deforestation likelihood can also control for variations in bird diversity indices (e.g., altitude and agricultural suitability – which includes climatic and topographic sites conditions – may correlate with endemism rate or species richness; remoteness may correlate with richness in threatened and Near Threatened species). Finally, local variations in species diversity that would not be covered by these expected relations are controlled for thanks to the spatial autocorrelation term that enables bird diversity indices to vary spatially and with seasons, independently from protection (see Supplementary Figures 10-17).

In summary, we expect intrinsic habitat differences between protected and non-protected sites to have been relatively minor in this study, and that both pre-existing differences in habitat or bird diversity and differences in surrounding pressure have been adequately controlled for in the statistical models.

E. Potential effect of protected area age on effectiveness

If protected area effects on biodiversity are due to implementation effects rather than location biases, it is expected that they increase with time. Effectiveness should therefore be higher in older protected areas. The World Database on Protected Areas includes a date for each protected area (status year; $status\_yr$) which can be used to test this hypothesis. Two caveats are however important to note: status year does not necessarily correspond to the year that given territory was first protected, but to the year of establishment of the current PA (page 40 in UNEP-WCMC[31]: *"if a Game Reserve designated in 1990 changed status to National Park in 2005, the status year for the National Park designation will be 2005 and the earlier Game Reserve will no longer be in the WDPA"*), which may mask a potential increase in effectiveness with protected area age. Second, whereas earlier protected areas were more frequently established to protect scenic landscapes or particular resources (e.g. game), recent decisions on protected area location are more likely to have incorporated better data on the distribution of, and threats to, biodiversity, including a stronger focus on threatened species (even because much of those data are themselves quite recent), and so protected areas are not necessarily expected to have had less impact over time.

We extracted for each protected site the status year of the protected area (we kept the lowest if several protected areas overlapped) and used linear models to study the effect of status year on the residuals of models from analyses I (i.e., the remaining difference in bird diversity indices that is not explained by protection, duration, expertise, latitude, longitude, remoteness, altitude, agricultural suitability).

This was modelled (for 8 hotspots * 4 bird indices) as: $residuals\_M1 \sim status\_year$ (8)

We then extracted linear coefficient and P-values, which are represented in Supplementary Figure 18.

A positive significant effect of age on residuals when the result of analysis I was positive (green bars pointing up, $N = 6$) suggests that old protected areas have higher residuals than young protected areas and then that they pulled the positive effect of analysis I more than young protected areas. A negative significant effect of age on residuals when the result of analysis I was negative (brown bars pointing down, $N = 3$) suggests that old protected areas have lower residuals than young protected areas and then that they pulled the negative effect of analysis I more than young protected areas. In both cases, this indicates that older protected areas performed better than younger ones, and thus a cumulative effect of protection over time. However most results are non-significant (either the effect of protected areas was non-significant in analysis 1 or the effect of status year on residuals was non-significant; $N = 22$) and one result was contrary to our expectation ($N = 1$, for threatened and Near Threatened species in IND).

Supplementary Tables

Supplementary Table 1: Effect of protected areas on each of the response variables considered in analyses I, II and IIIa, measured as percentage of difference. For analysis I, this was obtained by first predicting the response variable in a protected [$Resp_{In}$] and in an unprotected site [$Resp_{Out}$], while fixing all other variables to their median value. We then calculated the percentage of increase due to protection [$100 * (Resp_{In} – Resp_{Out})/abs(Resp_{Out})$], which estimates how richer an average site can be if protected rather than unprotected. We did the same for analysis IIIa, predicting the response variables in two unprotected sites, one forested [$Resp_{In}$] and one not forested [$Resp_{Out}$], with all variables fixed to their median values. For analysis II, we focused on background sites that are currently protected. We then predicted from our models response variables (probability of forest presence; each of the three habitat quality variables) for each site, first setting them as protected [$Resp_{PA}$], and second setting them as unprotected [$Resp_{unPA}$]. We then calculated the ratio percentage of increase due to protection [$100 * (Resp_{PA} – Resp_{unPA})/abs(Resp_{unPA})$] which estimates how much habitat loss or degradation would have happened had these sites not been protected. Column "MEAN" shows the average effect across the eight hotspots.

**ANALYSIS**	**RESPONSE VARIABLE**	ATL (%)	AND (%)	TUM (%)	MES (%)	EAS (%)	GHA (%)	IND (%)	SUN (%)	**Mean (%)**
**I**	Overall Richness	– 3.2	0.0	11.4	– 5.1	2.4	– 1.3	2.0	3.4	**1.7**
**I**	Forest-dependent	22.8	13.4	5.1	13.8	78.7	6.8	1.5	0.1	**17.8**
**I**	Endemic	18.9	24.0	– 24.2	7.1	635.4	– 25.5	– 6.4	– 8.6	**77.6**
**I**	Threatened and Near Threatened	17.1	33.7	7.3	37.1	– 22.0	27.7	58.9	– 7.5	**19.0**
**IIa**	Forest presence	51.6	3.6	1.7	4.2	32.9	18.1	20.2	10.1	**17.8**
**IIa'**	Deforestation rates	– 46.2	– 31.9	– 36.3	– 48.9	– 42.1	– 29.7	– 72.3	– 66.2	**– 46.7**
**IIb**	Canopy height	5.1	-0.5	15.0	3.1	3.8	-1.5	10.5	2.8	**4.8**
**IIb**	Forest contiguity	5.4	0.9	1.4	2.2	0.8	3.5	4.6	1.7	**2.6**
**IIb**	Wilderness	1.3	2.2	6.4	4.5	3.0	4.0	9.0	14.9	**5.7**
**IIIa**	Overall Richness	13.0	13.1	-1.8	17.3	-6.5	-14.2	-7.2	8.2	**2.7**
**IIIa**	Forest-dependent	97.3	126.0	35.9	53.0	153.8	43.2	53.8	36.1	**74.9**
**IIIa**	Endemic	266.9	109.2	18.2	30.5	1160.1	252.7	44.4	118.0	**250.0**
**IIIa**	Threatened and Near Threatened	246.7	152.0	101.8	227.1	-17.3	-19.6	63.0	223.1	**122.1**

Supplementary Table 2: Summary statistics per hotspot. Number of eBird checklists, observations, species and observers, after data selection (i.e., as used in the analyses). Average values for each of the four bird diversity indices considered: overall richness, richness in forest-dependent species, richness in endemics, and richness in threatened and Near Threatened species.

Hotspot name	Hotspot code	Number of checklists	Number of observations	Number of species	Number of observers	Richness (median ± se)	Richness in forest-dependent species (median ± se)	Richness in endemic species (median ± se)	Richness in threatened and NT species (median ± se)
Atlantic Forest	ATL	6,760	286,547	940	928	38 ± 26	19 ± 21	1 ± 4	1 ± 3
Tropical Andes	AND	17,758	683,213	2,229	2,244	34 ± 24	22 ± 19	4 ± 9	1 ± 2
Tumbes-Chocó-Magdalena	TUM	1,188	44,382	914	509	34 ± 21	16 ± 17	0 ± 0	1 ± 1
Mesoamerica	MES	32,784	1,363,889	1,185	3503	38 ± 24	24 ± 18	6 ± 6	1 ± 1
Eastern Afromontane	EAS	1,097	52,364	986	263	46 ± 25	11 ± 9	0 ± 0	0 ± 1
Western Ghats and Sri Lanka	GHA	2,646	99,567	487	556	37 ± 19	15 ± 11	0 ± 2	0 ± 1
Indo-Burma	IND	2,996	102,558	1,031	418	34 ± 17	10 ± 12	1 ± 2	0 ± 2
Sundaland	SUN	1,548	54,534	706	170	33 ± 19	15 ± 16	2 ± 11	1 ± 5

Supplementary Table 3: Statistical power of each test made in analyses I, as the number of checklists used in each test (N), the P-value of the test (P), and the R-squared of the test (R^2).

Hotspot name	Hotspot code	Nb of checklists	Overall richness		Forest-dependent		Endemic		Thr. and Near Thr.	
			P	R^2 (%)	P	R^2 (%)	P	R^2 (%)	P	R^2 (%)
Atlantic Forest	ATL	6,760	0.025	44.8	$8.0.10^{-81}$	85.4	$1.4.10^{-12}$	73.5	$4.7.10^{-9}$	69.2
Tropical Andes	AND	17,758	0.984	50.5	$3.9.10^{-111}$	89.2	$1.1.10^{-53}$	69.4	$1.9.10^{-44}$	46.7
Tumbes-Chocó-Magdalena	TUM	1,188	0.016	41.1	0.022	91.6	0.268	30.4	0.344	49.9
Mesoamerica	MES	32,784	$9.8.10^{-15}$	46.3	0	88.7	$3.3.10^{-43}$	80.2	$7.8.10^{-125}$	39.8
Eastern Afromontane	EAS	1,097	0.566	70.8	$6.6.10^{-30}$	77.8	$6.1.10^{-4}$	65.0	0.153	45.4
Western Ghats and Sri Lanka	GHA	2,646	0.680	42.6	0.018	79.4	0.016	74.8	0.0131	39.0
Indo-Burma	IND	2,996	0.398	47.2	0.558	80.5	0.225	70.9	$1.1.10^{-8}$	70.0
Sundaland	SUN	1,548	0.507	41.9	0.978	92.8	0.086	89.4	0.223	89.9

Supplementary Figures

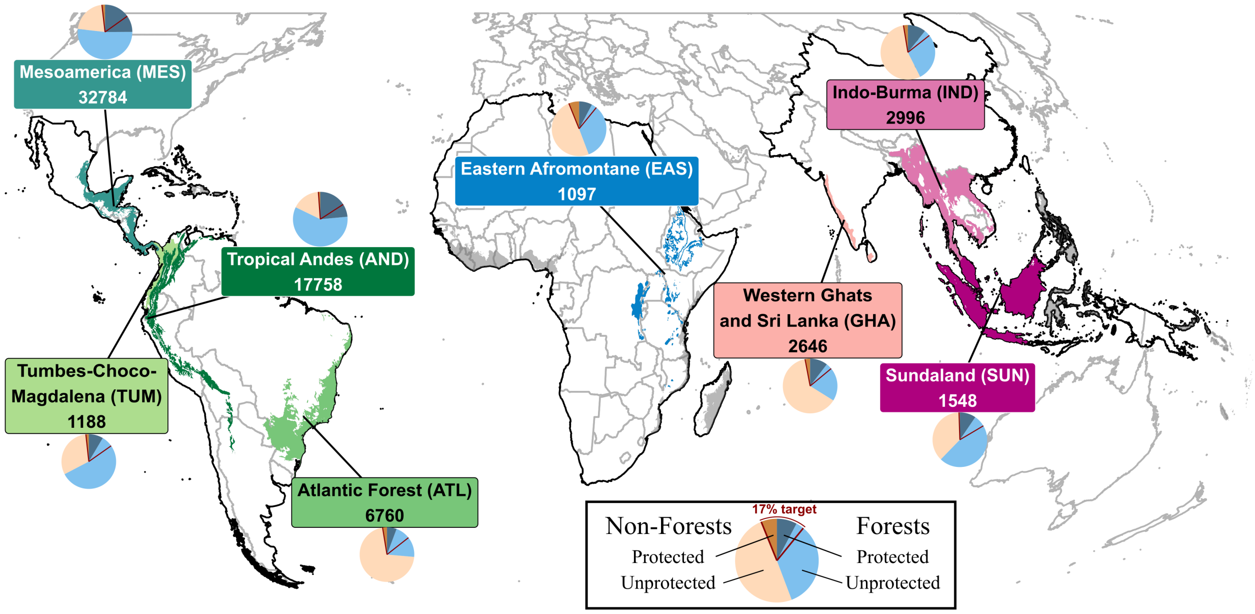

Supplementary Figure 1: Regions covered by the present study, with number of checklists and levels of protection for each hotspot. Coloured regions correspond to the area analysed within each of the eight hotspots, i.e. the intersection between the hotspot boundary and the "tropical and subtropical moist broadleaf forests" biome. For these, boxes indicate the hotspot name, acronym, and number of checklists analysed. Pie plots represent the proportion of forest/non-forest and protected/non-protected background sites in each hotspot. Red lines in pie plots show the 17% protection target: when the right line falls within light blue, protection of the study region is < 17%. Gray regions in the map correspond to other forest hotspots considered for analysis but with less than 1000 eBird checklists. The black lines in the map indicate the limits of each continent used to calculate observer experience and expertise.

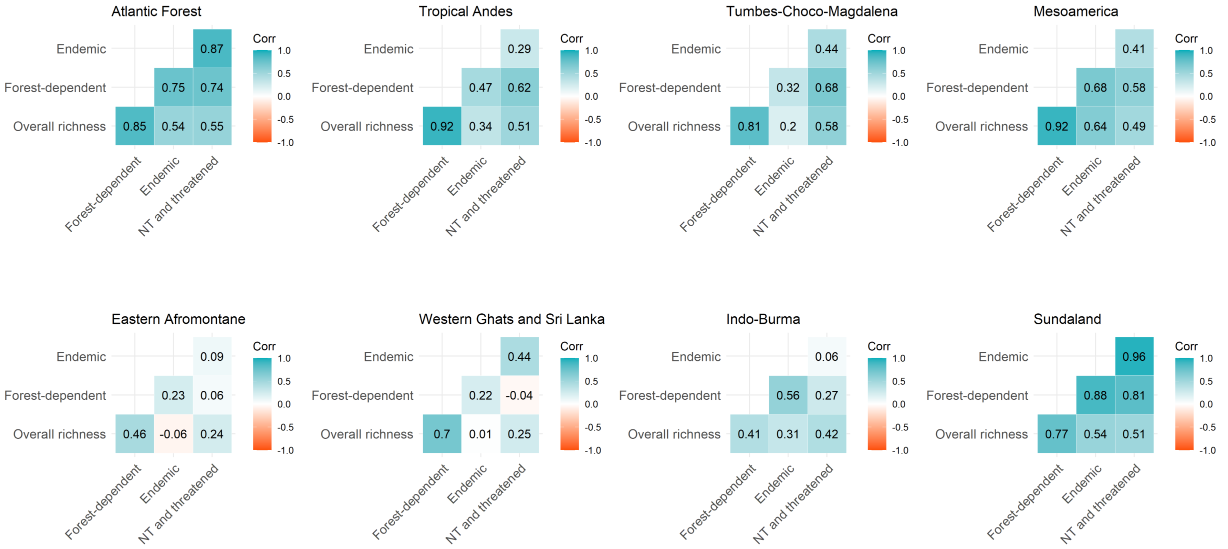

Supplementary Figure 2: Correlation between bird diversity indices (overall richness, richness in forest-dependent species, richness in endemic species, and richness in threatened and Near Threatened species) across hotspots. These correlations, when high, could explain the results consistency across bird diversity indices. However, they do not compromise the results as we are not looking for a causal relation between bird diversity indices and protected areas effects (e.g., are species effectively conserved by protected areas **because** they are endemic species) but we are interested in species of conservation concern in their own rights (e.g., are endemic species effectively conserved by protected areas).

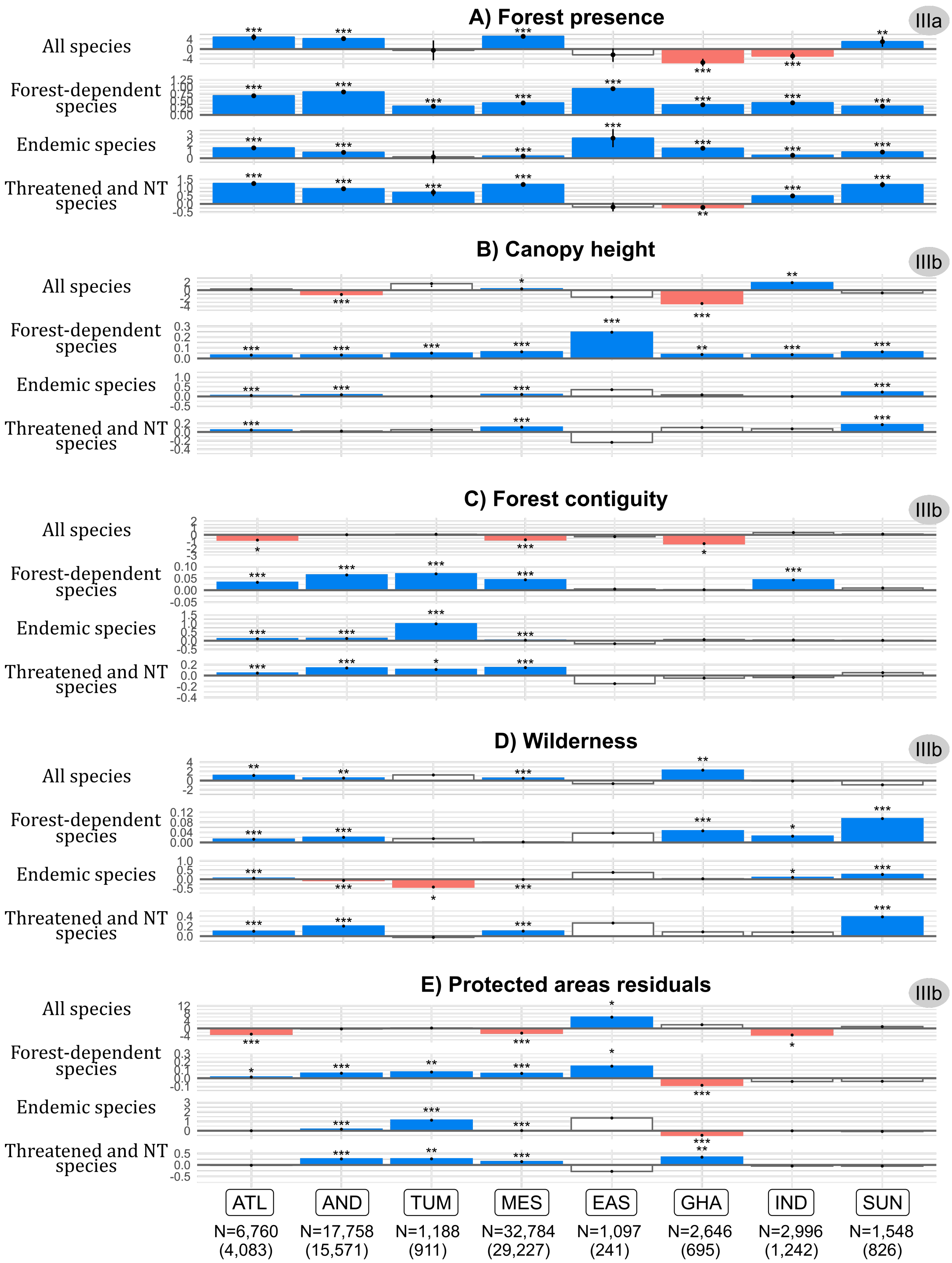

Supplementary Figure 3: Effects of forest presence and forest quality on bird diversity per hotspot. Analysis IIIa (A) shows the effect of forest presence on bird diversity. Analysis IIIb shows the effect of forest quality (B, Canopy height; C, Forest contiguity; D, Wilderness; E, Protected areas residuals) on bird diversity. Bird diversity is measured through four indices of richness in: all species, forest-dependent species, endemic species, threatened and Near Threatened species. Coefficients correspond to the estimates of GAM models; significance given by P-value ($* * * < 0.001 < ** < 0.10 < * < 0.05$), and 95% confidence interval around GAM coefficients (vertical error bars). Hotspots: ATL (Atlantic Forest), AND (Tropical Andes), TUM (Tumbes-Chocó-Magdalena), MES (Mesoamerica), EAS (Eastern Afromontane), GHA (Western Ghats and Sri Lanka), IND (Indo-Burma), SUN (Sundaland). Number checklists used per hotspots are given below hotspots names: number of checklists used in analysis IIIa (i.e., forest and non-forest checklists) first, and number of checklists used in analysis IIIb (i.e., only forest checklists) in brackets.

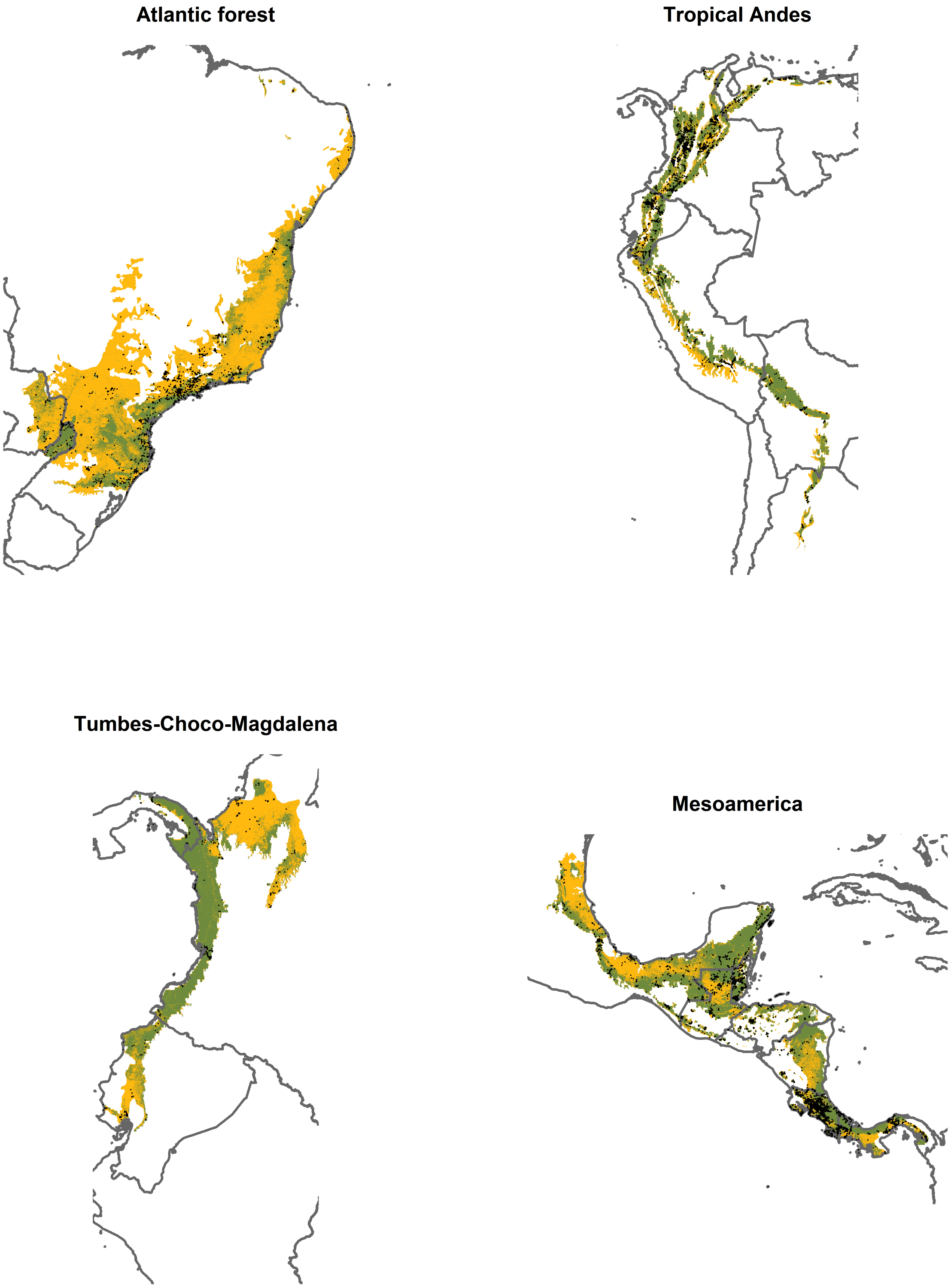

Supplementary Figure 4: Extent and sampling effort within each of the hotspot in the Americas. Each map indicates the full extent of the area analysed (i.e., the intersection between the hotspot and the "tropical and subtropical moist broadleaf forests" biome). Green represents forest, orange other habitats. Black dots represent the locations of the checklists used in the analyses.

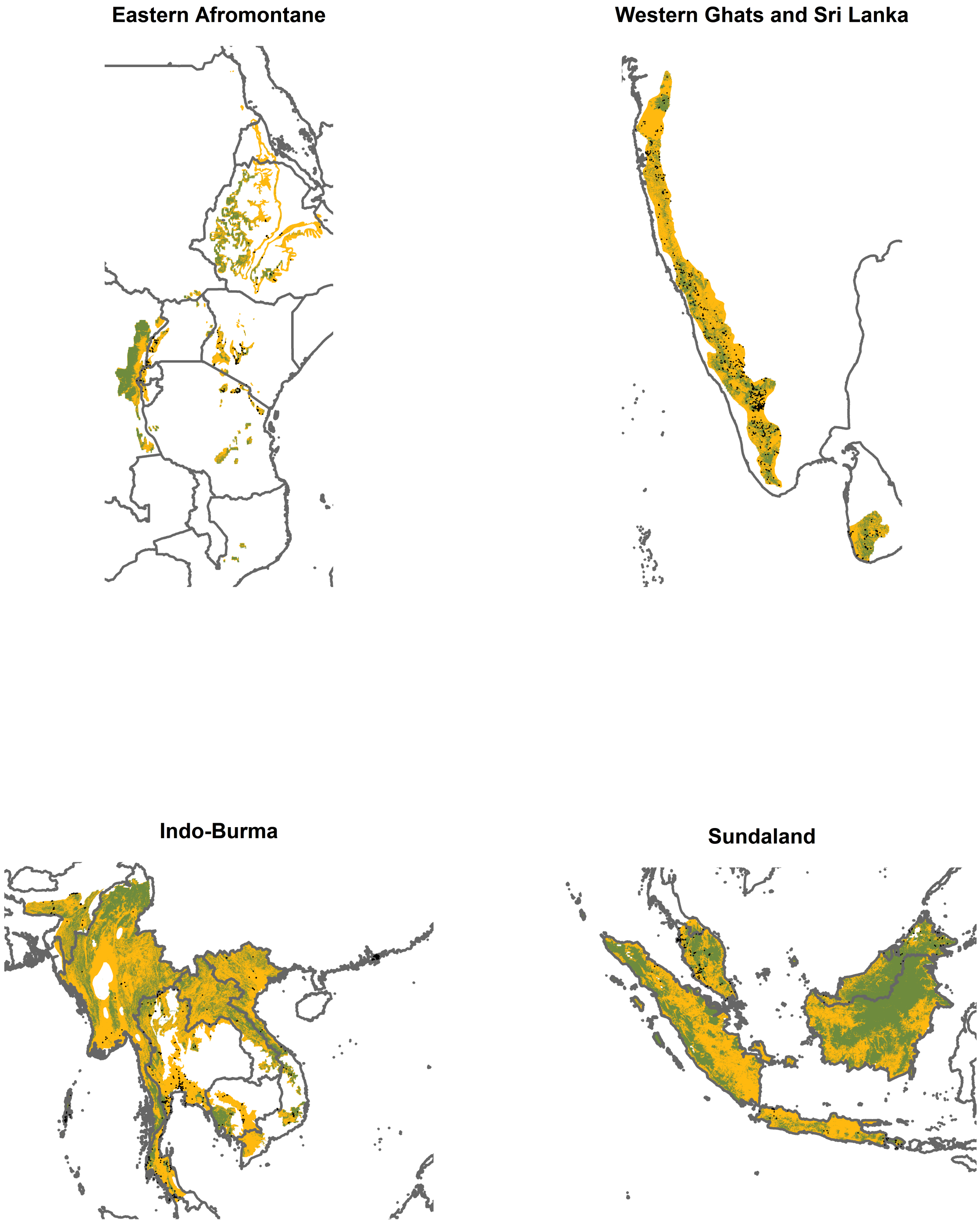

Supplementary Figure 5: Extent and sampling effort within each of the hotspot in Africa and Asia. Each map indicates the full extent of the area analysed (i.e., the intersection between the hotspot and the "tropical and subtropical moist broadleaf forests" biome). Green represents forest, orange other habitats. Black dots represent the locations of the checklists used in the analyses.

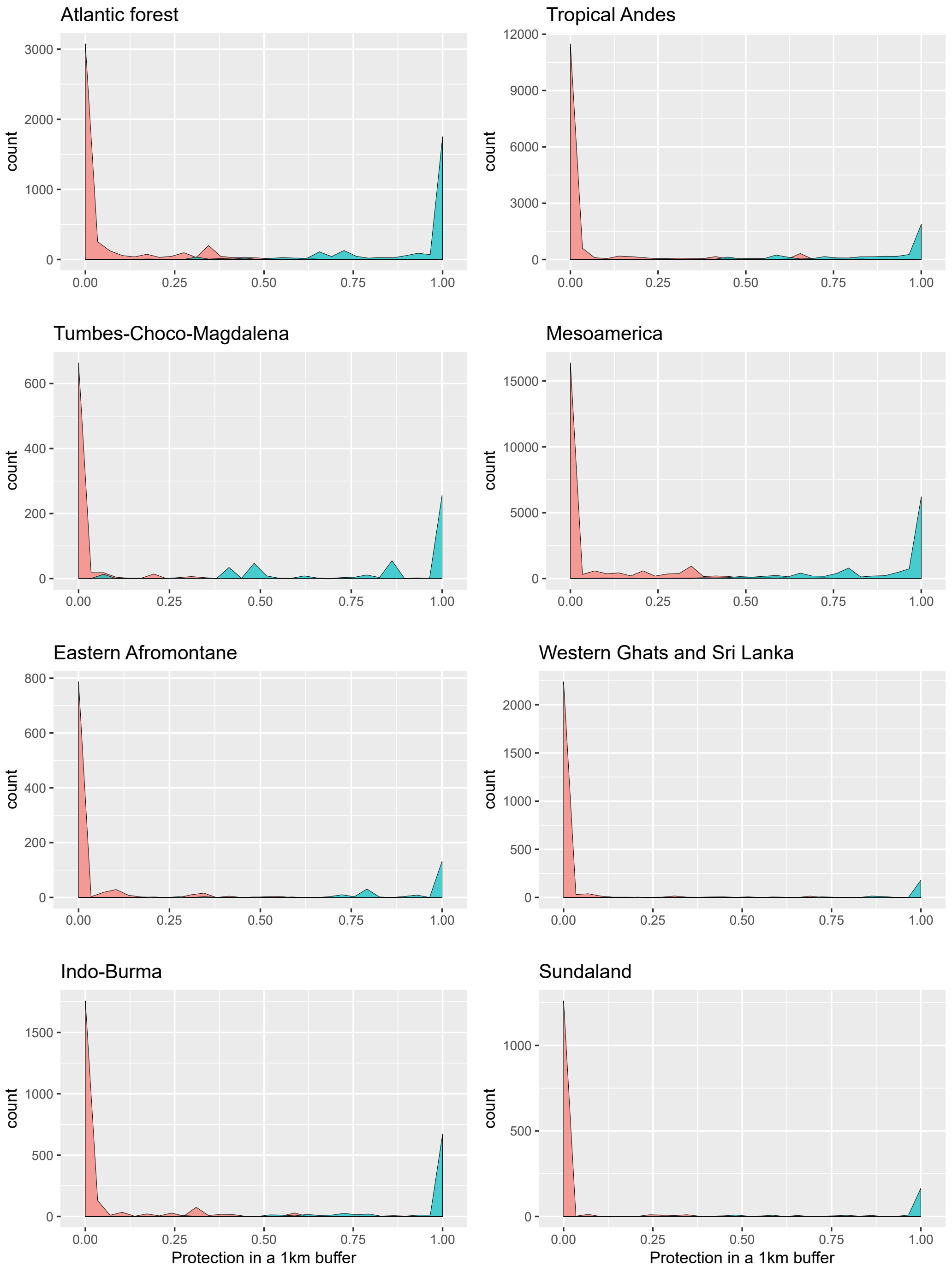

Supplementary Figure 6: Comparison between a binary classification of protection based on the protection status of the site coordinates (red for unprotected, blue for protected sites) as used in the analyses, and a continuous classification by measuring the proportion of area protected within a 1km buffer around each site.

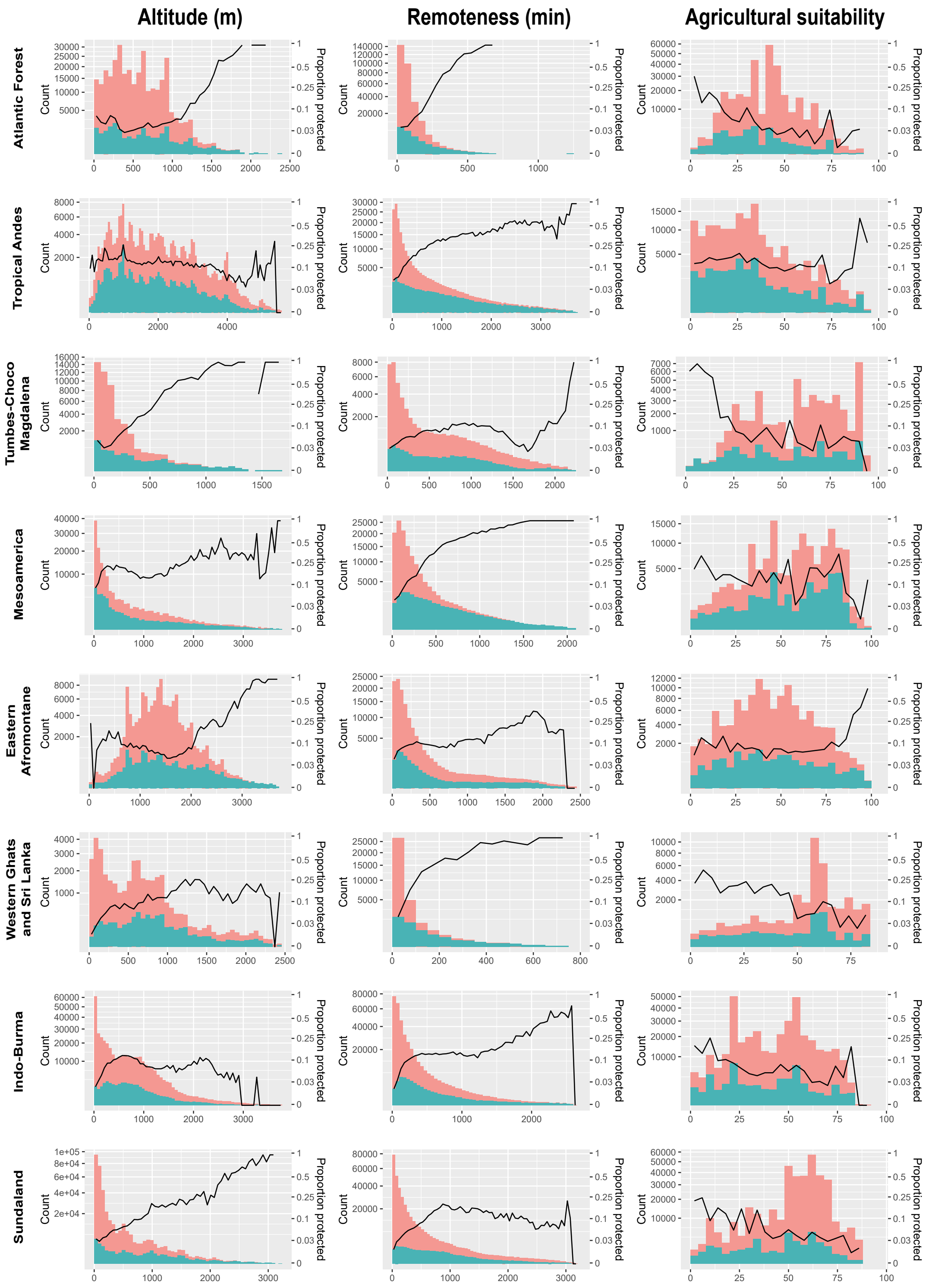

Supplementary Figure 7: Distribution of three variables used to control for location bias (altitude, remoteness, and agricultural suitability) for protected and unprotected sites (respectively blue and red bars) and proportion of sites protected for each bar (black lines), per hotspot. y-scales are transformed through square root function.

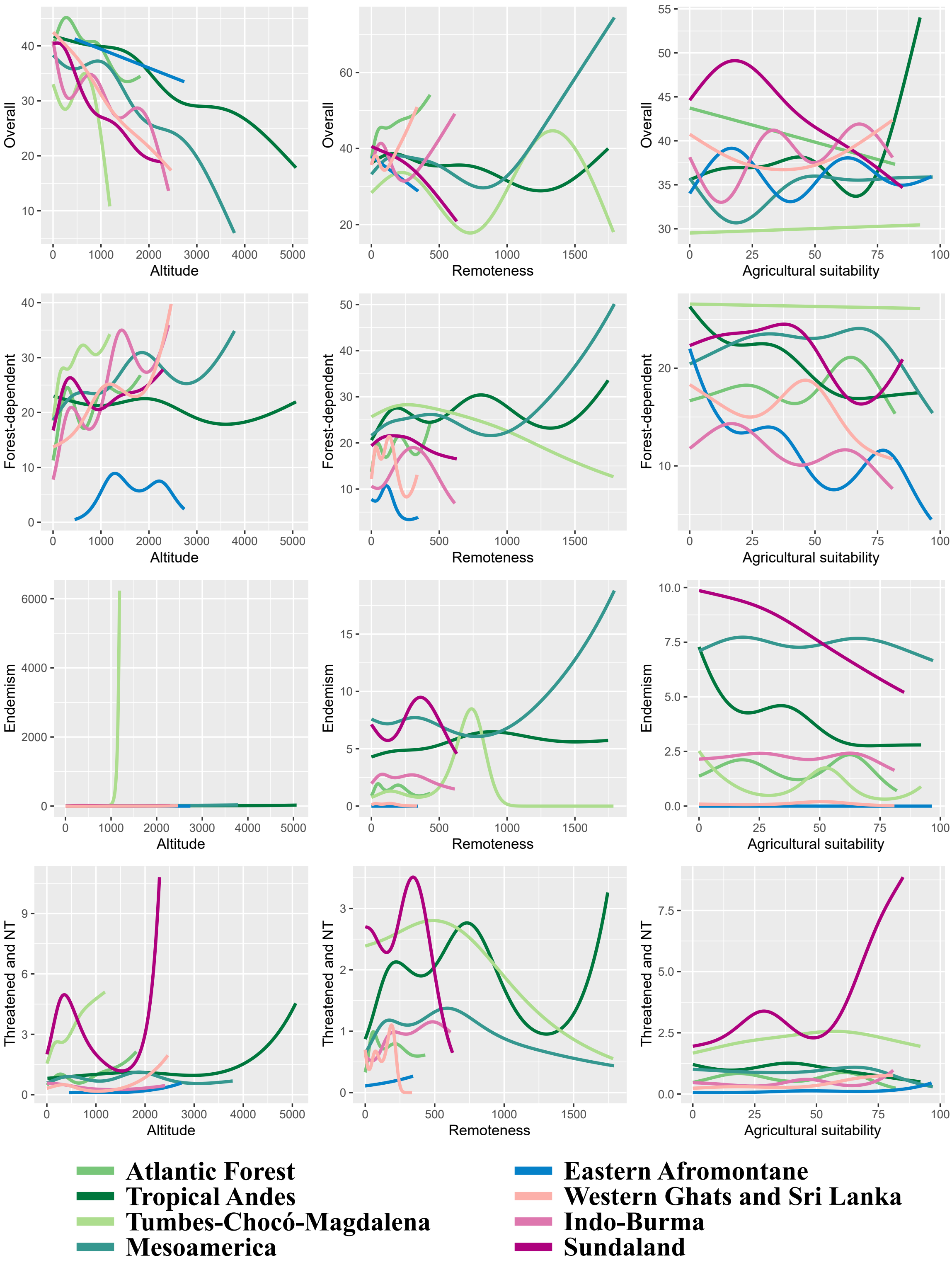

Supplementary Figure 8: Effects of each of the variables used to control for location bias in analysis I (altitude, remoteness and agricultural suitability) on each of the indices of bird diversity (overall species richness, richness in forest-dependent species, richness in endemic species, and richness in threatened and Near Threatened species), for each hotspot. Effects were predicted fixing all other variables to their median values and the protection variable to "unprotected".

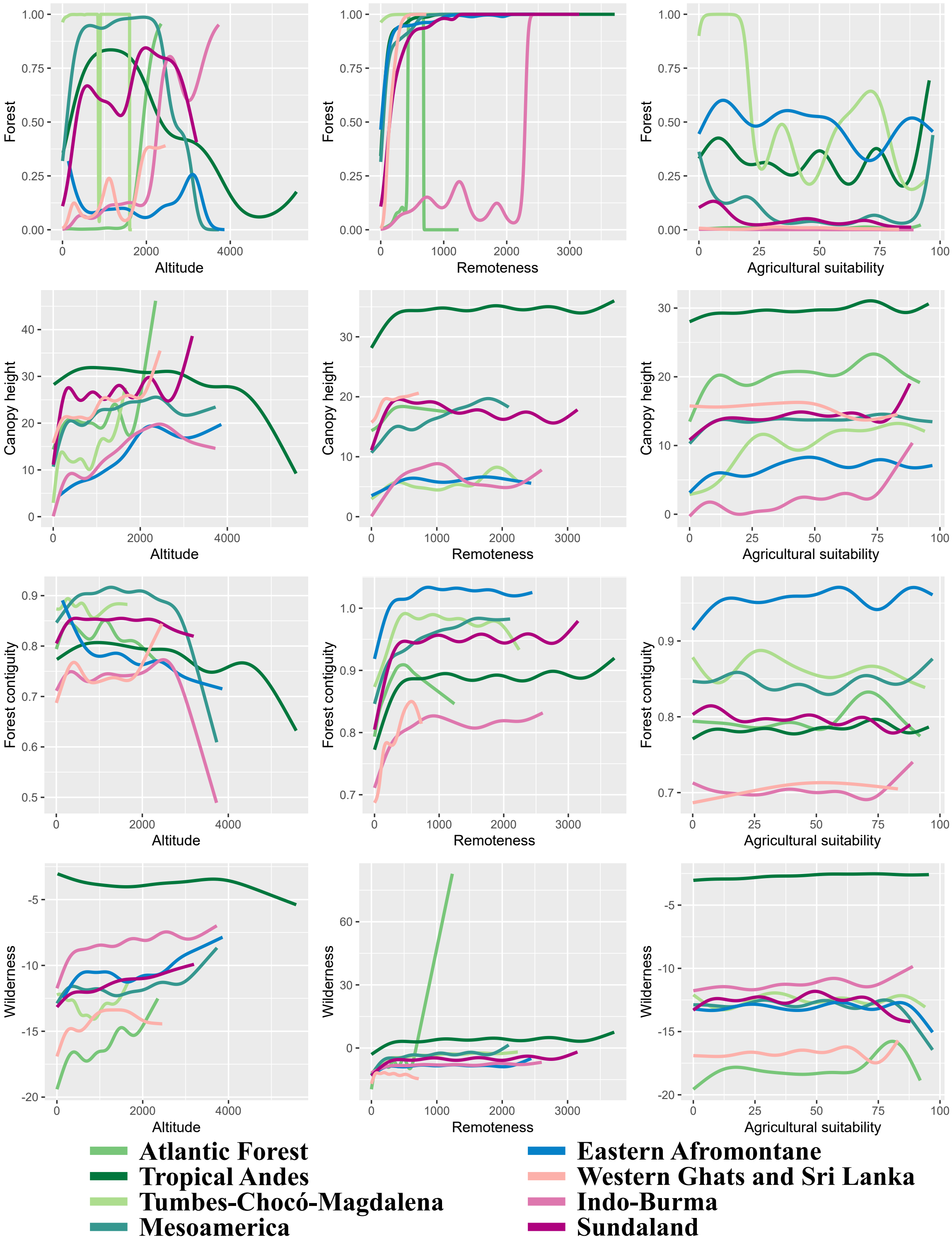

Supplementary Figure 9: Effects of each of the variables used to control for location bias in analysis II (altitude, remoteness and agricultural suitability) on forest presence and on each of the indices of forest quality (canopy height, forest contiguity, wilderness), for each hotspot. Effects were predicted fixing all other variables to their median values and the protection variable to “unprotected”.

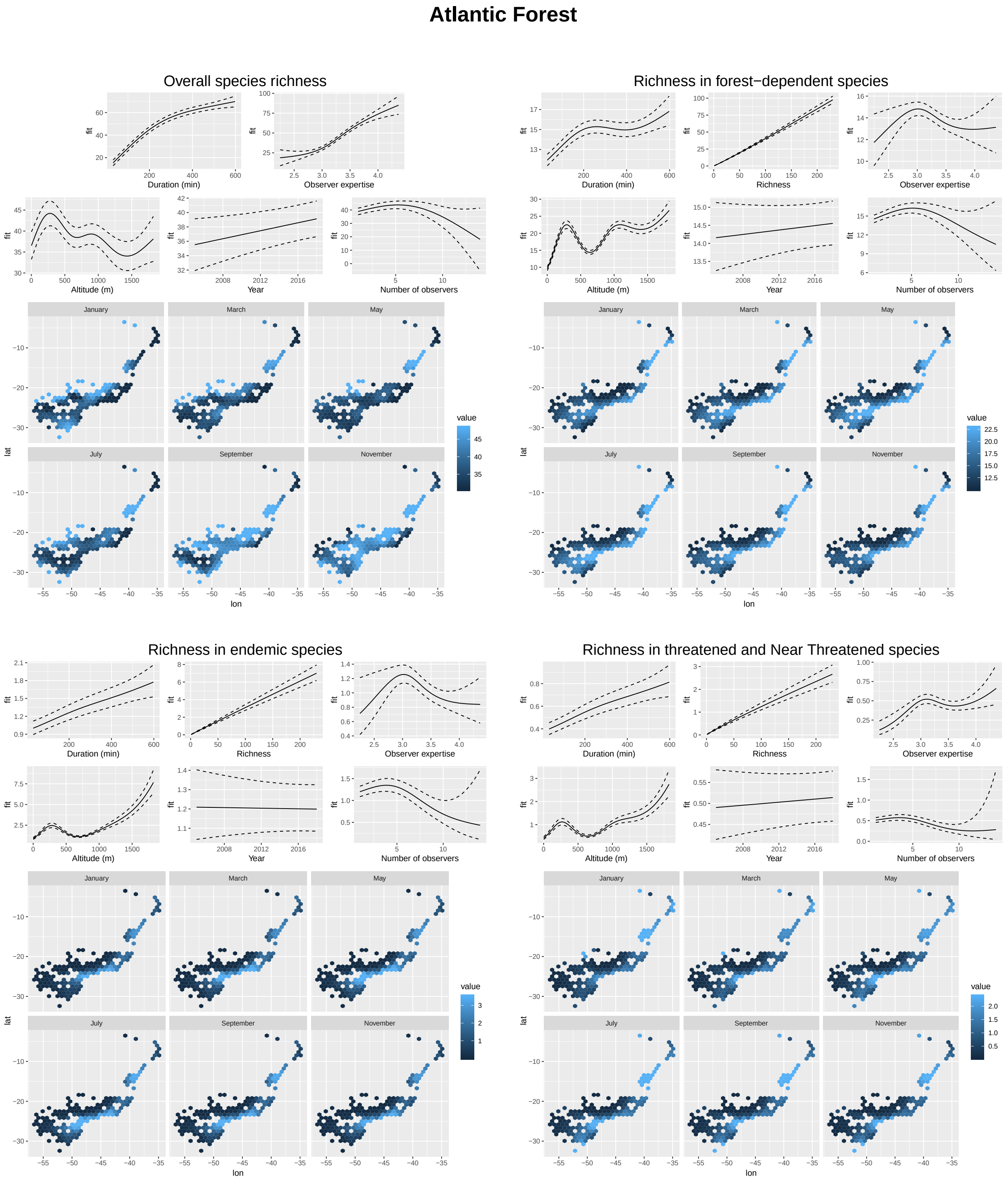

Supplementary Figure 10: Effects of each of the covariates used as controls in analysis III on each of the four bird diversity indices (overall species richness, richness in forest-dependent species, richness in endemic species, and richness in threatened and Near Threatened species), for the Atlantic Forest hotspot. We predicted bird indices (i.e., y values are always number of species) fixing all other variables to their median values. Maps represent spatial variation and seasonality for each diversity index. They correspond to a predict of each bird diversity index obtained by making longitude and latitude vary across the hotspot and fixing other variables to their median values, for 6 dates (mid-January [day 15], mid-March [day 74], mid-May [day 135], mid-July [day 196], mid-September [day 258], mid-November [day 319]), smoothed on a hexagonal grid by the ggplot function *stat_summary_hex* with default settings.

Tropical Andes

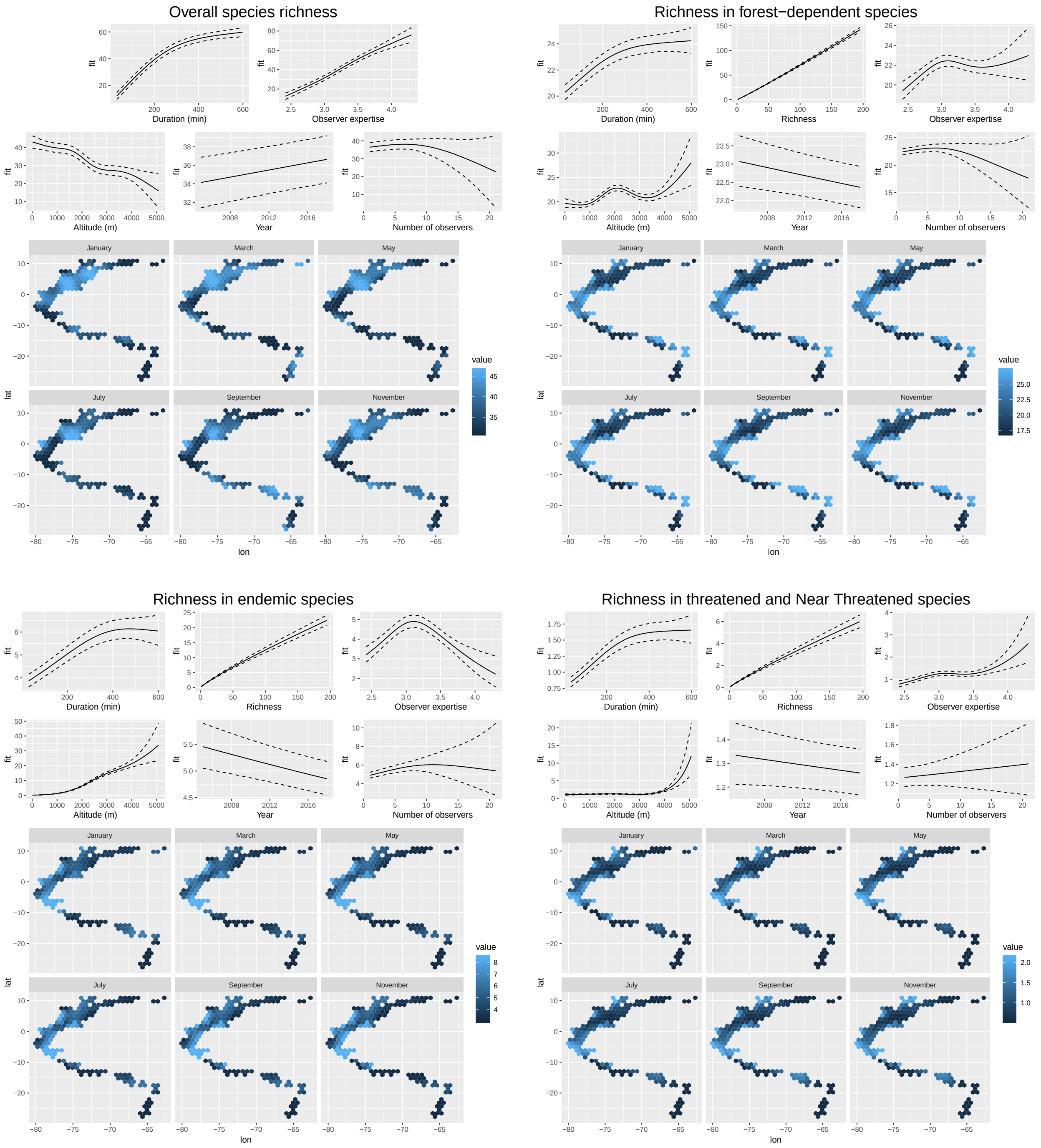

Supplementary Figure 11: Effects of each of the covariates used as controls in analysis III on each of the four bird diversity indices (overall species richness, richness in forest-dependent species, richness in endemic species, and richness in threatened and Near Threatened species), for the Tropical Andes hotspot. We predicted bird indices (i.e., y values are always number of species) fixing all other variables to their median values. Maps represent spatial variation and seasonality for each diversity index. They correspond to a predict of each bird diversity index obtained by making longitude and latitude vary across the hotspot and fixing other variables to their median values, for 6 dates (mid-January [day 15], mid-March [day 74], mid-May [day 135], mid-July [day 196], mid-September [day 258], mid-November [day 319]), smoothed on a hexagonal grid by the ggplot function *stat_summary_hex* with default settings.

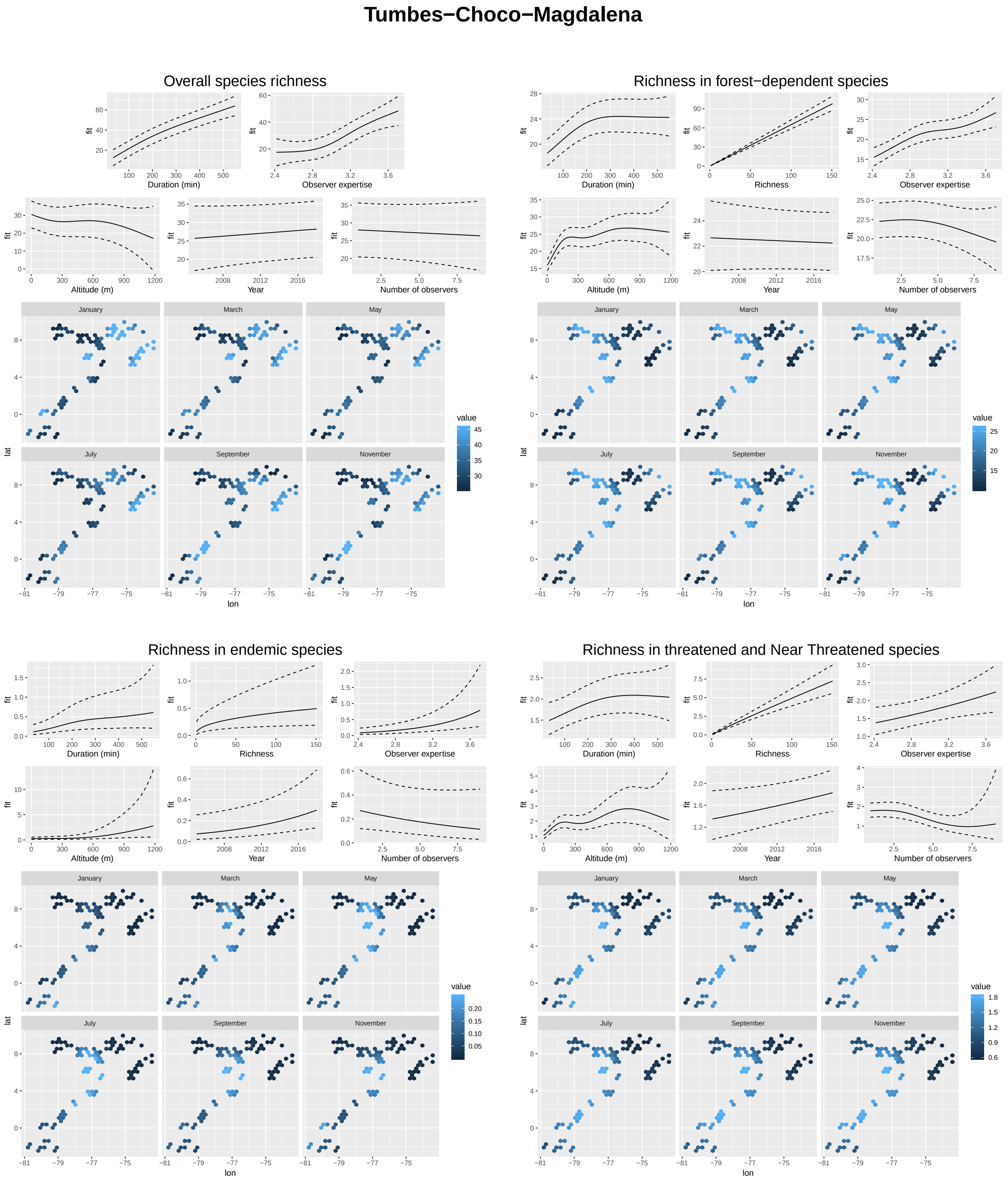

Supplementary Figure 12: Effects of each of the covariates used as controls in analysis III on each of the four bird diversity indices (overall species richness, richness in forest-dependent species, richness in endemic species, and richness in threatened and Near Threatened species), for the Tumbes-Choco-Magdalena hotspot. We predicted bird indices (i.e., y values are always number of species) fixing all other variables to their median values. Maps represent spatial variation and seasonality for each diversity index. They correspond to a predict of each bird diversity index obtained by making longitude and latitude vary across the hotspot and fixing other variables to their median values, for 6 dates (mid-January [day 15], mid-March [day 74], mid-May [day 135], mid-July [day 196], mid-September [day 258], mid-November [day 319]), smoothed on a hexagonal grid by the ggplot function *stat_summary_hex* with default settings.

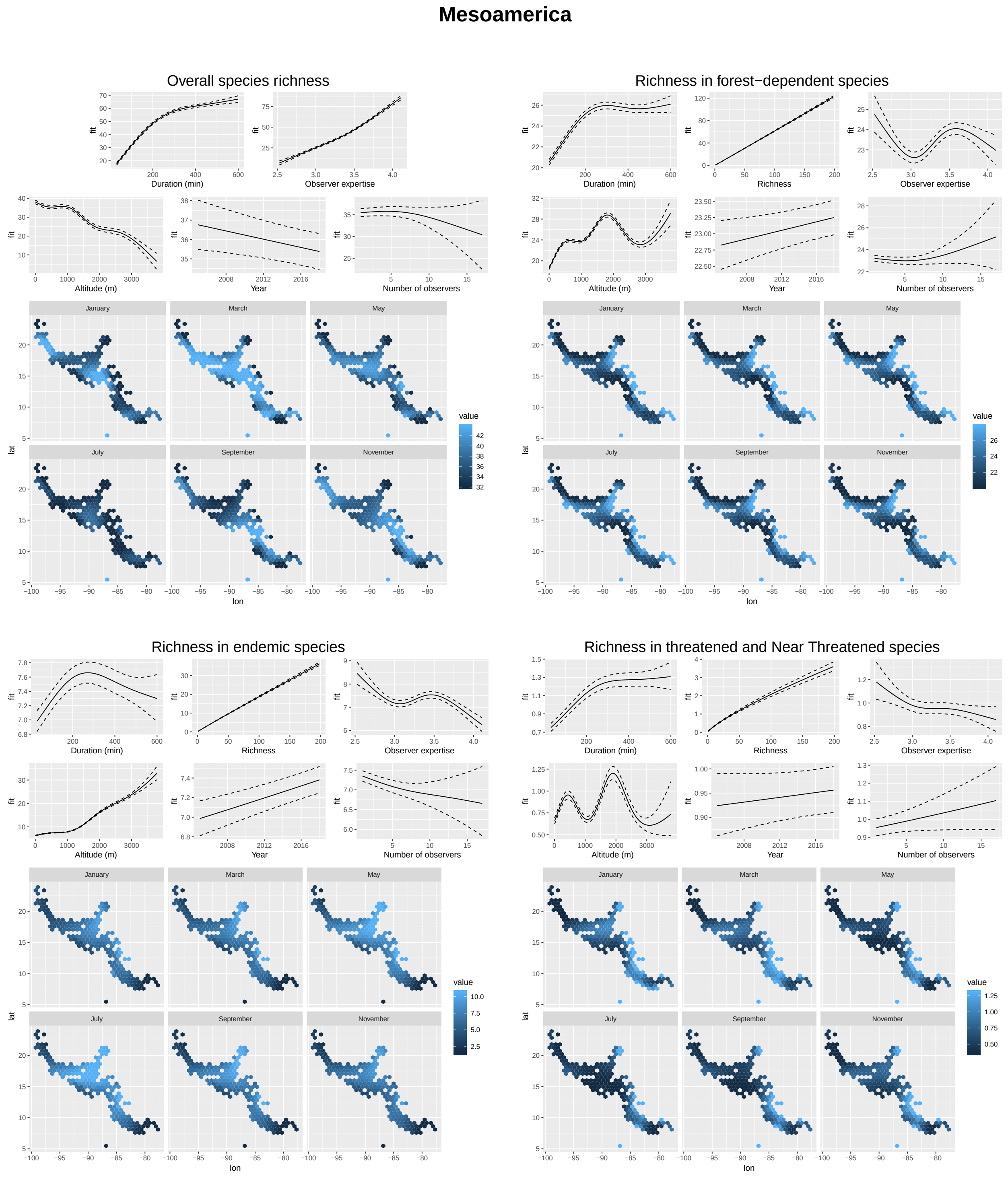

Supplementary Figure 13: Effects of each of the covariates used as controls in analysis III on each of the four bird diversity indices (overall species richness, richness in forest-dependent species, richness in endemic species, and richness in threatened and Near Threatened species), for the Mesoamerica hotspot. We predicted bird indices (i.e., y values are always number of species) fixing all other variables to their median values. Maps represent spatial variation and seasonality for each diversity index. They correspond to a predict of each bird diversity index obtained by making longitude and latitude vary across the hotspot and fixing other variables to their median values, for 6 dates (mid-January [day 15], mid-March [day 74], mid-May [day 135], mid-July [day 196], mid-September [day 258], mid-November [day 319]), smoothed on a hexagonal grid by the ggplot function *stat_summary_hex* with default settings.

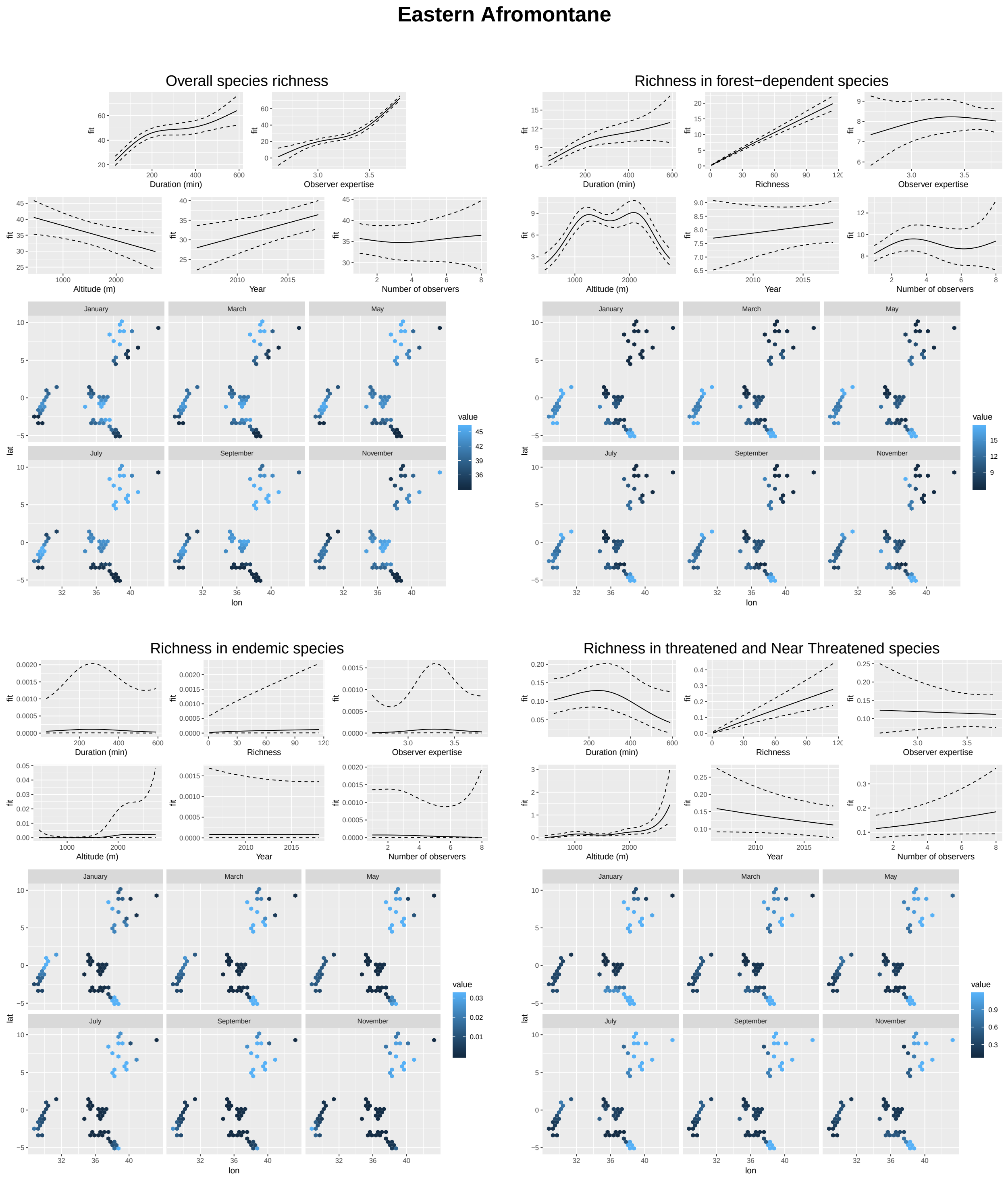

Supplementary Figure 14: Effects of each of the covariates used as controls in analysis III on each of the four bird diversity indices (overall species richness, richness in forest-dependent species, richness in endemic species, and richness in threatened and Near Threatened species), for the Eastern Afromontane hotspot. We predicted bird indices (i.e., y values are always number of species) fixing all other variables to their median values. Maps represent spatial variation and seasonality for each diversity index. They correspond to a predict of each bird diversity index obtained by making longitude and latitude vary across the hotspot and fixing other variables to their median values, for 6 dates (mid-January [day 15], mid-March [day 74], mid-May [day 135], mid-July [day 196], mid-September [day 258], mid-November [day 319]), smoothed on a hexagonal grid by the ggplot function *stat_summary_hex* with default settings.

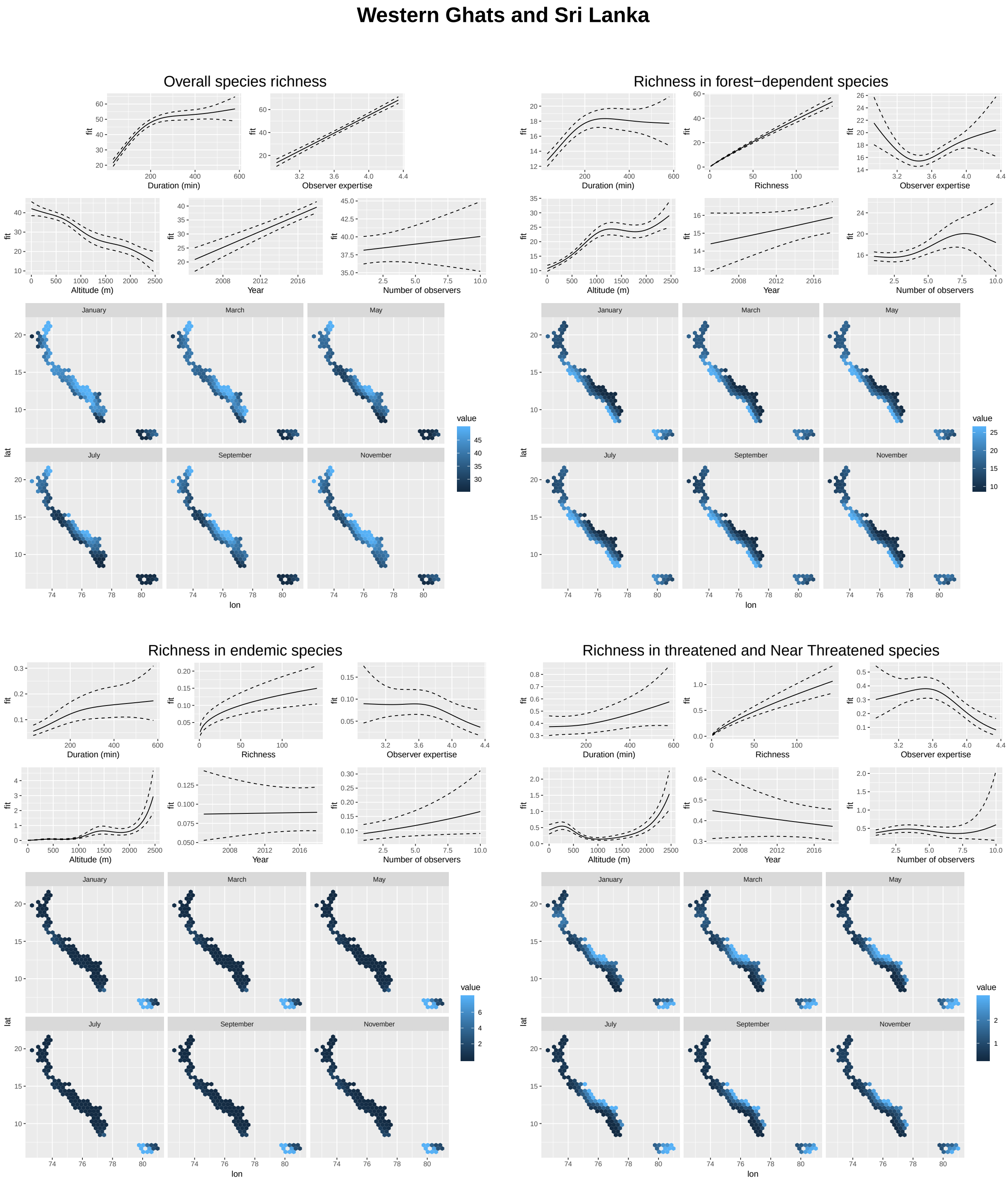

Supplementary Figure 15: Effects of each of the covariates used as controls in analysis III on each of the four bird diversity indices (overall species richness, richness in forest-dependent species, richness in endemic species, and richness in threatened and Near Threatened species), for the Western Ghats and Sri Lanka hotspot. We predicted bird indices (i.e., y values are always number of species) fixing all other variables to their median values. Maps represent spatial variation and seasonality for each diversity index. They correspond to a predict of each bird diversity index obtained by making longitude and latitude vary across the hotspot and fixing other variables to their median values, for 6 dates (mid-January [day 15], mid-March [day 74], mid-May [day 135], mid-July [day 196], mid-September [day 258], mid-November [day 319]), smoothed on a hexagonal grid by the ggplot function *stat_summary_hex* with default settings.

Indo−Burma

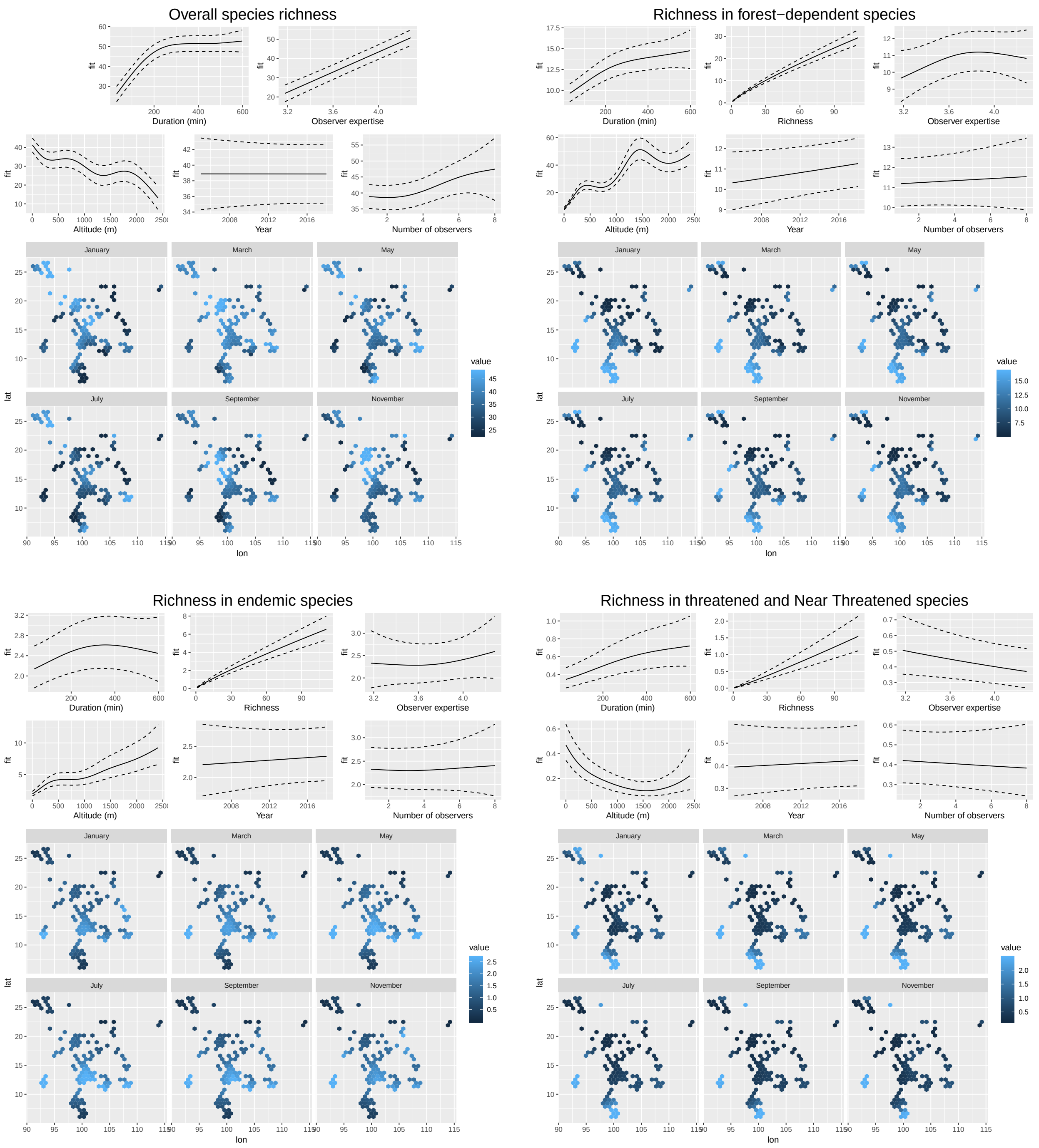

Supplementary Figure 16: Effects of each of the covariates used as controls in analysis III on each of the four bird diversity indices (overall species richness, richness in forest-dependent species, richness in endemic species, and richness in threatened and Near Threatened species), for the Indo-Burma hotspot. We predicted bird indices (i.e., y values are always number of species) fixing all other variables to their median values. Maps represent spatial variation and seasonality for each diversity index. They correspond to a predict of each bird diversity index obtained by making longitude and latitude vary across the hotspot and fixing other variables to their median values, for 6 dates (mid-January [day 15], mid-March [day 74], mid-May [day 135], mid-July [day 196], mid-September [day 258], mid-November [day 319]), smoothed on a hexagonal grid by the ggplot function *stat_summary_hex* with default settings.

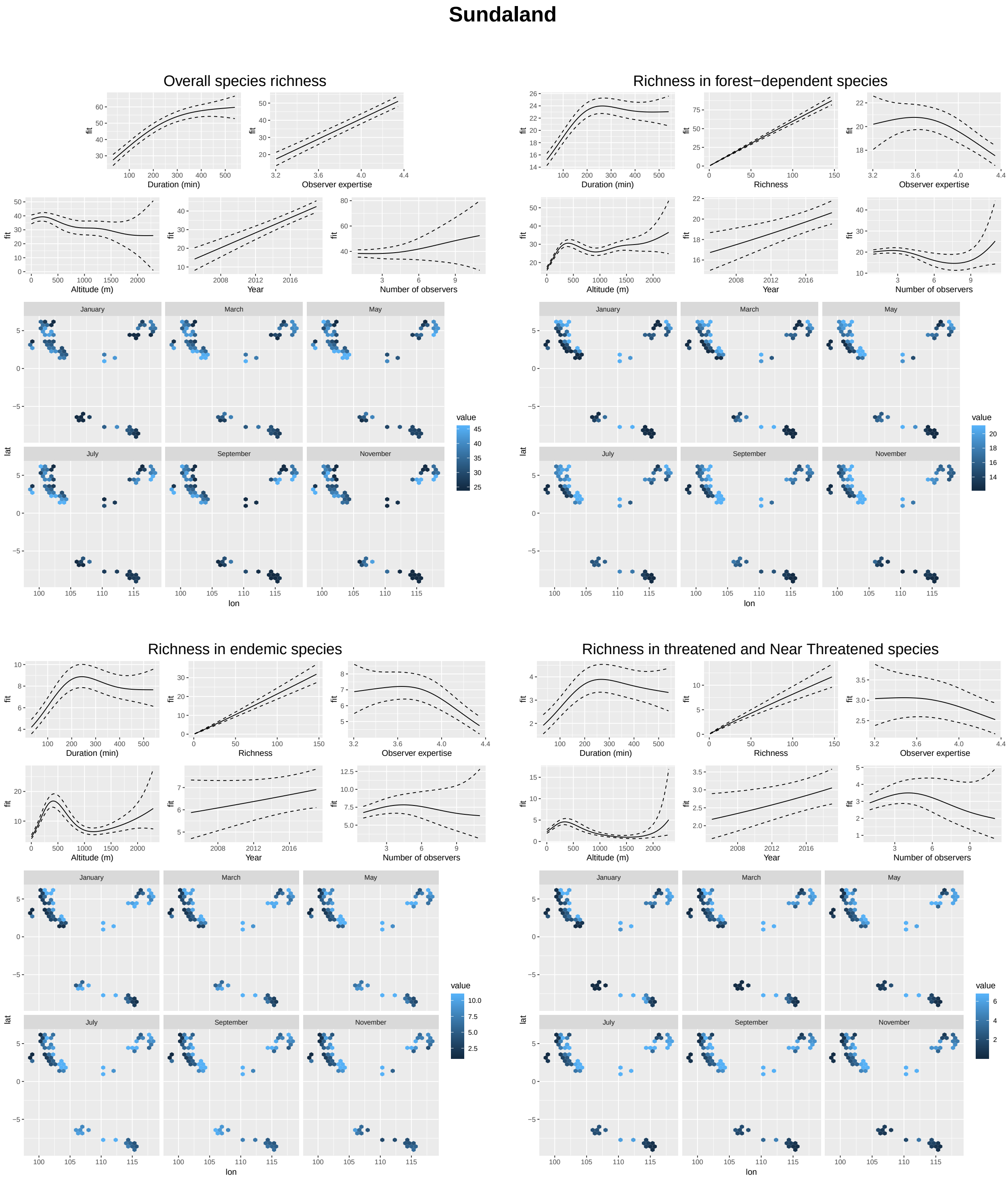

Supplementary Figure 17: Effects of each of the covariates used as controls in analysis III on each of the four bird diversity indices (overall species richness, richness in forest-dependent species, richness in endemic species, and richness in threatened and Near Threatened species), for the Sundaland hotspot. We predicted bird indices (i.e., y values are always number of species) fixing all other variables to their median values. Maps represent spatial variation and seasonality for each diversity index. They correspond to a predict of each bird diversity index obtained by making longitude and latitude vary across the hotspot and fixing other variables to their median values, for 6 dates (mid-January [day 15], mid-March [day 74], mid-May [day 135], mid-July [day 196], mid-September [day 258], mid-November [day 319]), smoothed on a hexagonal grid by the ggplot function *stat_summary_hex* with default settings.

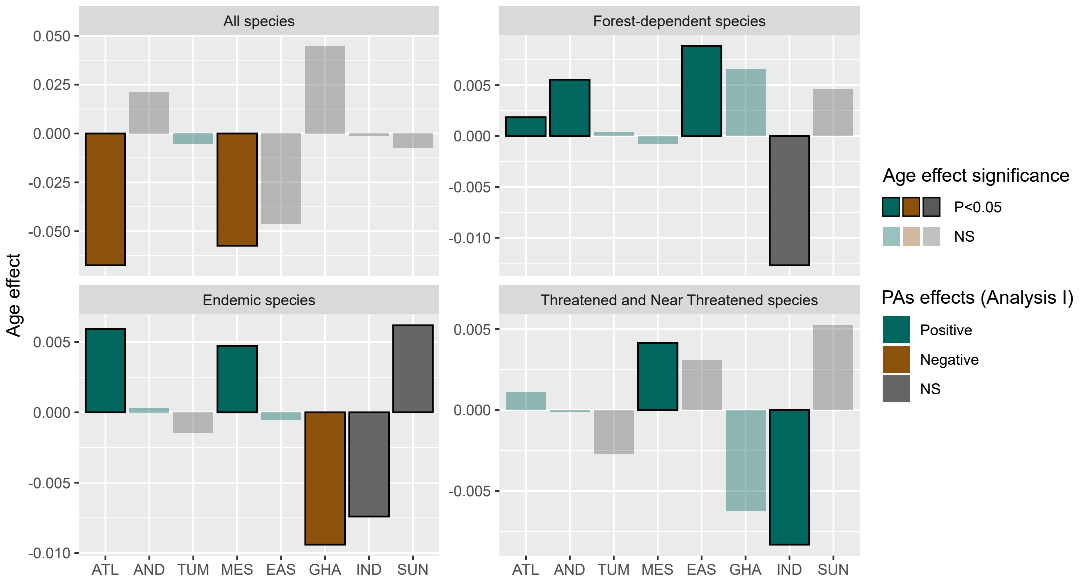

Supplementary Figure 18: Effect of protected areas age (i.e., 2020 - year of creation or last change in status of protected areas) on residuals of models in analysis I. Bar colours recall results of Analysis I (testing for the effects of protected areas on bird diversity). Dark shades indicate statistical significance for the effect of protected areas age. A positive significant effect of age on residuals when the result of analysis I was positive (green bars pointing up, N=6) suggests that old protected areas have higher residuals than young protected areas and then that they pulled the positive effect of analysis I more than young protected areas. A negative significant effect of age on residuals when the result of analysis I was negative (brown bars pointing down, N=3) suggests that old protected areas have lower residuals than young protected areas and then that they pulled the negative effect of analysis I more than young protected areas. In both cases, this indicates that older protected areas performed better than younger ones, and thus a cumulative effect of protection over time. However most results are non-significant (either the effect of protected areas was non-significant in analysis 1 or the effect of status year on residuals was non-significant; N=22) and one result was contrary to our expectation (N=1, for threatened and Near Threatened species in IND).

Supplementary Discussion

When comparing across hotspots, we found some heterogeneity in our results, potentially induced by three factors. First, failure to comply with our simplifying hypothesis that our study regions (i.e., extent of biodiversity hotspots included in the "tropical and subtropical moist broadleaf forests" biome) were originally covered by homogeneous forest could explain some differences in bird responses. For instance, responses of bird diversity indices in Western Ghats and Sri Lanka were often low, sometimes the opposite of others. This could be due to the natural habitat heterogeneity of this region, such as the presence of natural grasslands above the shola forests and large variations in rainfall patterns[1]; this may also be the case in the Tropical Andes, which include high natural grasslands such as Páramo (see Supplementary Methods 4D for further information on this assumption), and perhaps Eastern Afromontane. This corroborates with the high proportion of species with null dependency of forest in Western Ghats and Sri Lanka, as well as Eastern Afromontane (respectively 33% and 38% of species detected, against an average of 18% for other hotspots). Second, differences in protection regimes could explain some of the variation found between hotspots. Indeed, the location of protected areas in the Atlantic Forest is not highly biased towards remote and high areas (reactive approach[32], Supplementary Figure 7), which could explain the high effectiveness measured. Conversely, protected areas in Sundaland are highly biased towards remote and high zones that are less likely to suffer from human pressure in the short-term (pro-active approach[32], Supplementary Figure 7), which could explain the low effect we measured while controlling for location biases. Finally, sampling effort greatly differed between hotspots (ranging from 1,070 checklists analysed in Eastern Afromontane to 31,053 in Mesoamerica; see Supplementary Table 3), which could affect the statistical power of tests. This could explain why hotspots in the Americas showed clearer results than those from Asia and Africa, particularly for the effects of protected areas in maintaining forest quality. The consistency of results we got across continents in analyses II and III (disentangling the mechanisms of protected area effects on bird diversity) give high credit to this assumption.

References

[1] Russell A. Mittermeier. Hotspot revisited.

[2] David M. Olson, Eric Dinerstein, Eric D. Wikramanayake, Neil D. Burgess, George V. N. Powell, Emma C. Underwood, Jennifer A. D'amico, Illanga Itoua, Holly E. Strand, John C. Morrison, Colby J. Loucks, Thomas F. Allnutt, Taylor H. Ricketts, Yumiko Kura, John F. Lamoreux, Wesley W. Wettengel, Prashant Hedao, and Kenneth R. Kassem. Terrestrial ecoregions of the world: A new map of life on EarthA new global map of terrestrial ecoregions provides an innovative tool for conserving biodiversity. 51(11):933–938.

[3] UNEP-WCMC and IUCN. Protected planet: [WDPA-shapefile-polygons; the world database on protected areas (WDPA)/the global database on protected areas management effectiveness (GD-PAME)] [on-line, downloaded 02/10/2018], cambridge, UK. <www.protectedplanet.net>.

[4] Brian L. Sullivan, Christopher L. Wood, Marshall J. Iliff, Rick E. Bonney, Daniel Fink, and Steve Kelling. eBird: A citizen-based bird observation network in the biological sciences. 142(10):2282–2292.

[5] Matthew Strimas-Mackey, Eliot Miller, and Wesley Hochachka. *auk: eBird Data Extraction and Processing with AWK*.

[6] J.F. Clements, T.S. Schulenberg, M.J. Iliff, D. Roberson, T.A. Fredericks, B.L. Sullivan, and C.L. Wood. The eBird/clements checklist of birds of the world: v2018 [downloaded from <http://www.birds.cornell.edu/clementschecklist/download/>].

[7] BirdLife International and HBW. Bird species distribution maps of the world. version 7.0. available at <http://datazone.birdlife.org/species/requestdis>.

[8] UNEP-WCMC and IUCN. Calculating protected area coverage. [on-line, consulted 06/02/2019]. <www.protectedplanet.net/c/calculating-protected-area-coverage>.

[9] ESA. Climate change initiative - land cover project map v2.0.7. data from year 2015. <http://maps.elie.ucl.ac.be/CCI/viewer/index.php>.

[10] National Geophysical Data Center. Global land one-kilometer base elevation (GLOBE), version 1. <https://www.ngdc.noaa.gov/mgg/topo/gltiles.html>.

[11] Florian Zabel, Birgitta Putzenlechner, and Wolfram Mauser. Global agricultural land resources – a high resolution suitability evaluation and its perspectives until 2100 under climate change conditions. 9(9).

[12] D. J. Weiss, A. Nelson, H. S. Gibson, W. Temperley, S. Peedell, A. Lieber, M. Hancher, E. Poyart, S. Belchior, N. Fullman, B. Mappin, U. Dalrymple, J. Rozier, T. C. D. Lucas, R. E. Howes, L. S. Tusting, S. Y. Kang, E. Cameron, D. Bisanzio, K. E. Battle, S. Bhatt, and P. W. Gething. A global map of travel time to cities to assess inequalities in accessibility in 2015. 553(7688):333–336.

[13] Marc Simard, Naiara Pinto, Joshua B. Fisher, and Alessandro Baccini. Mapping forest canopy height globally with spaceborne lidar. 116.

[14] Oscar Venter, Eric W. Sanderson, Ainhoa Magrach, James R. Allan, Jutta Beher, Kendall R. Jones, Hugh P. Possingham, William F. Laurance, Peter Wood, Balázs M. Fekete, Marc A. Levy, and James E.M. Watson. Global terrestrial human footprint maps for 1993 and 2009. 3:160067.

[15] Andrew J. Hansen and Ruth DeFries. Ecological mechanisms linking protected areas to surrounding lands. 17(4):974–988.

[16] BirdLife International. IUCN red list for birds. version 2017.1. downloaded from <http://www.birdlife.org>.

[17] Steve Kelling, Alison Johnston, Wesley M. Hochachka, Marshall Iliff, Daniel Fink, Jeff Gerbracht, Carl Lagoze, Frank A. La Sorte, Travis Moore, Andrea Wiggins, Weng-Keen Wong, Chris Wood, and Jun Yu. Can observation skills of citizen scientists be estimated using species accumulation curves? 10(10):e0139600.

[18] Alison Johnston, Daniel Fink, Wesley M. Hochachka, and Steve Kelling. Estimates of observer expertise improve species distributions from citizen science data. 9(1):88–97.

[19] Simon N. Wood. Fast stable restricted maximum likelihood and marginal likelihood estimation of semiparametric generalized linear models: Estimation of semiparametric generalized linear models. 73(1):3–36.

[20] Alain F. Zuur, E.N. Ieno, N. Walker, A.A. Saveliev, and G.M. Smith. *Mixed effects models and extensions in ecology with R*. Statistics for biology and health. Springer. OCLC: 288985460.

[21] A Nelson and K.M. Chomitz. Protected area effectiveness in reducing tropical deforestation.

[22] Navin Ramankutty and Jonathan A. Foley. Estimating historical changes in global land cover: Croplands from 1700 to 1992. 13(4):997–1027.

[23] IBGE - EMBRAPA. Mapa de solos do brasil. rio de janeiro - escala 1:5.000.000. the original vegetation map (shapefile format) can be downloaded for brazil or brazilian legal amazon limits.

[24] Jaclyn Hall, Neil D. Burgess, Jon Lovett, Boniface Mbilinyi, and Roy E. Gereau. Conservation implications of deforestation across an elevational gradient in the eastern arc mountains, tanzania. 142(11):2510–2521.

[25] Kwaw S. Andam, Paul J. Ferraro, Alexander Pfaff, G. Arturo Sanchez-Azofeifa, and Juan A. Robalino. Measuring the effectiveness of protected area networks in reducing deforestation. 105(42):16089–16094.

[26] Pablo Cuenca, Rodrigo Arriagada, and Cristian Echeverría. How much deforestation do protected areas avoid in tropical andean landscapes? 56:56–66.

[27] Jonas Geldmann, Andrea Manica, Neil D. Burgess, Lauren Coad, and Andrew Balmford. A global-level assessment of the effectiveness of protected areas at resisting anthropogenic pressures. 116(46):23209–23215.

[28] Oscar Venter, Ainhoa Magrach, Nick Outram, Carissa Joy Klein, Hugh P. Possingham, Moreno Di Marco, and James E. M. Watson. Bias in protected-area location and its effects on long-term aspirations of biodiversity conventions. 32(1):127–134.

[29] Germán Baldi, Marcos Texeira, Osvaldo A. Martin, H. Ricardo Grau, and Esteban G. Jobbágy. Opportunities drive the global distribution of protected areas. 5:e2989.

[30] Laura J. Pollock, Wilfried Thuiller, and Walter Jetz. Large conservation gains possible for global biodiversity facets. 546(7656):141–144.

[31] UNEP-WCMC. User manual for the world database on protected areas and world database on other effective area-based conservation measures:1.6. available at: http://wcmc.io/WDPA_manua.

[32] T. M. Brooks, R. A. Mittermeier, G. A. B. da Fonseca, J. Gerlach, M. Hoffmann, J. F. Lamoreux, C. G. Mittermeier, J. D. Pilgrim, and A. S. L. Rodrigues. Global biodiversity conservation priorities. 313(5783):58–61.
