# Supplementary material for: Effectiveness of protected areas in conserving tropical forest birds
Source: Nat Commun. 2020 Sep 14;11:4461. doi: 10.1038/s41467-020-18230-0 (PMC7490714; doi:10.1038/s41467-020-18230-0)
Supplement: Supplementary file 3 — Reporting Summary [file 41467_2020_18230_MOESM3_ESM.pdf]

nature research

Corresponding author(s): Victor Cazalis

Last updated by author(s): Jul 24, 2020

Reporting Summary

Nature Research wishes to improve the reproducibility of the work that we publish. This form provides structure for consistency and transparency in reporting. For further information on Nature Research policies, see Authors & Referees and the Editorial Policy Checklist .

Statistics

For all statistical analyses, confirm that the following items are present in the figure legend, table legend, main text, or Methods section.

n/a	Confirmed	
☐	☒	The exact sample size (n) for each experimental group/condition, given as a discrete number and unit of measurement
☒	☐	A statement on whether measurements were taken from distinct samples or whether the same sample was measured repeatedly
☐	☒	The statistical test(s) used AND whether they are one- or two-sided<br>*Only common tests should be described solely by name; describe more complex techniques in the Methods section.*
☐	☒	A description of all covariates tested
☒	☐	A description of any assumptions or corrections, such as tests of normality and adjustment for multiple comparisons
☐	☒	A full description of the statistical parameters including central tendency (e.g. means) or other basic estimates (e.g. regression coefficient) AND variation (e.g. standard deviation) or associated estimates of uncertainty (e.g. confidence intervals)
☐	☒	For null hypothesis testing, the test statistic (e.g. F, t, r) with confidence intervals, effect sizes, degrees of freedom and P value noted<br>*Give P values as exact values whenever suitable.*
☒	☐	For Bayesian analysis, information on the choice of priors and Markov chain Monte Carlo settings
☒	☐	For hierarchical and complex designs, identification of the appropriate level for tests and full reporting of outcomes
☒	☐	Estimates of effect sizes (e.g. Cohen's d, Pearson's r), indicating how they were calculated

Our web collection on statistics for biologists contains articles on many of the points above.

Software and code

Policy information about availability of computer code

Data collection	No software was used for data collection.
Data analysis	We used R (version 3.6.0) for all analyses. All code newly developped to run these analyses (coded by the lead author) is available from: http://doi.org/10.5281/zenodo.3952401. In addition, we used the auk R package (version 0.3.2) and the mgcv R package (version 1.8-28).

For manuscripts utilizing custom algorithms or software that are central to the research but not yet described in published literature, software must be made available to editors/reviewers. We strongly encourage code deposition in a community repository (e.g. GitHub). See the Nature Research guidelines for submitting code & software for further information.

Data

Policy information about availability of data

All manuscripts must include a data availability statement. This statement should provide the following information, where applicable:

- Accession codes, unique identifiers, or web links for publicly available datasets
- A list of figures that have associated raw data
- A description of any restrictions on data availability

The data that support the findings of this study are all freely available for research purposes, and can either be directly downloaded from the respective website or are available on demand.

• Bird species checklists (eBird v. 12/2018), http://ebird.org;
• Bird species distribution maps (version V7.0), http://datazone.birdlife.org (available on demand);
• Bird species IUCN Red List status (IUCN Red List of Threatened Species, version 2017.1), http://iucnredlist.org;
• Protected area polygons (World Database on Protected Areas, v. 10/2018), https://www.protectedplanet.net/;
• Elevation data (GLOBE Digital Elevation Model, v.1, 1999), https://www.ngdc.noaa.gov/mgg/topo/gltiles.html;
• Biodiversity hotspots, https://www.cepf.net/our-work/biodiversity-hotspots/hotspots-defined;
• Biome boundaries, https://www.worldwildlife.org/publications/terrestrial-ecoregions-of-the-world;

• Forest cover (Climate Change Initiative Land cover data project map from 2015 v.2.0.7) http://maps.elie.ucl.ac.be/CCI/viewer/index.php;
• Agricultural suitability 1981-2010 (version 1.0), https://www.ufz.de/glues/;
• Remoteness (Accessibility to Cities, version 1.0), https://malariaatlas.org/research-project/accessibility_to_cities/;
• Canopy height (version 1.0), https://landscape.jpl.nasa.gov/;
• Human footprint (version 1.0), https://doi.org/10.5061/dryad.052q5;
• Forest loss (2000 to 2019, version 2019-v1.7), http://earthenginepartners.appspot.com/science-2013-global-forest.

Field-specific reporting

Please select the one below that is the best fit for your research. If you are not sure, read the appropriate sections before making your selection.

☐ Life sciences ☐ Behavioural & social sciences ☒ Ecological, evolutionary & environmental sciences

For a reference copy of the document with all sections, see nature.com/documents/nr-reporting-summary-flat.pdf

Ecological, evolutionary & environmental sciences study design

All studies must disclose on these points even when the disclosure is negative.

Study description	We compare bird occurrence records in protected versus unprotected sites in eight biodiversity hotspots across the tropics in order to measure protected areas effectiveness at conserving bird diversity. We then measure whether an effect of protected areas at mitigating forest loss and forest degradation can explain those results.
Research sample	The study is based on existing data on bird occurrence: checklists compiled through the eBird citizen science platform (version released in December 2018). Here, we analysed a total of 66,777 checklists that met the following conditions: collected between 2005 and 2018; within the study area (eight biodiversity hotspots analysed, restricted to their moist forest parts: Atlantic Forest, Tropical Andes, Tumbes-Chocó-Magdalena, Mesoamerica, Eastern Afromontane, Western Ghats and Sri Lanka, Indo-Burma, Sundaland); complete checklists only (i.e., in which observers explicitly declare having reported all bird species detected and identified); following either the 'stationary points' or the 'travelling counts' protocol; with durations of continuous observation of 0.5-10 hrs; with observers travelling distances during the checklist < 5 km; only from relatively experienced observers (≥ 10 checklists; ≥ 30 species per checklist on average; ≥ 100 different species in total). We removed potential duplicates (checklists made on the same day at the same place).
Sampling strategy	All data that meet the above mentioned conditions were included in the analyses.
Data collection	This study builds from previously existing data on bird occurrences. These data were collected in the form of checklists by volunteer observers (who also reported information on sampling effort) and then compiled and managed by the eBird team at the Cornell Lab of Ornithology.
Timing and spatial scale	Data were collected locally (points or transects < 5km), with observers providing GPS coordinates and timing of sampling sites. We considered only data from eight tropical biodiversity hotspots, between 2005 and 2018.
Data exclusions	We focused on records from 2005 to 2018, as data collected prior to 2005 were too scarce for analysis. We filtered the dataset based on the above-mentioned criteria (Research Sample) in order to obtain high-quality checklists comparable in protocol and effort. We removed potential duplicates (checklists made on the same day at the same place) to avoid pseudo-replication. We excluded hotspots for which we did not have sufficient data (<= 1000 checklists, after the above-mentioned data selection).
Reproducibility	All the data filtering and processing method is detailed, which should allow the reproductibility of this study's results. There was no experimental replication of this work as it is based on citizen science data analyses, not experiments.
Randomization	We did not apply any randomisation as we addressed sampling biases using statistical controls for sampling effort (following guidelines by eBird project managers)
Blinding	Blinding is not relevant for this study because it is an observational study (citizen science monitoring), and no experiments were carried out.

Did the study involve field work? ☐ Yes ☒ No

Reporting for specific materials, systems and methods

We require information from authors about some types of materials, experimental systems and methods used in many studies. Here, indicate whether each material, system or method listed is relevant to your study. If you are not sure if a list item applies to your research, read the appropriate section before selecting a response.

Materials & experimental systems

n/a	Involved in the study
☒	☐ Antibodies
☒	☐ Eukaryotic cell lines
☒	☐ Palaeontology
☒	☐ Animals and other organisms
☒	☐ Human research participants
☒	☐ Clinical data

Methods

n/a	Involved in the study
☒	☐ ChIP-seq
☒	☐ Flow cytometry
☒	☐ MRI-based neuroimaging
