# Supplementary material for: Effectiveness of protected areas in conserving tropical forest birds
Source: Nat Commun. 2020 Sep 14;11:4461. doi: 10.1038/s41467-020-18230-0 (PMC7490714; doi:10.1038/s41467-020-18230-0)
Supplement: Supplementary file 4 — Description of Additional Supplementary Files [file 41467_2020_18230_MOESM4_ESM.pdf]

Description of Additional Supplementary Files

File Name: Supplementary Data 1
Description: Detailed mismatches between eBird and BirdLife International taxonomies enabling to transform eBird data into BirdLife International taxonomy. The sheet "Change_Names" gives the species for which we only changed the eBird species name by the BirdLife International name. The sheet "Birdlife_lump" includes species that are considered as split in eBird taxonomy but are lumped in the BirdLife International taxonomy, for which we lumped eBird observations. The sheet "eBird_lump" includes species that are considered as lumped in eBird taxonomy but are split in the BirdLife International taxonomy, for which we split eBird observations based on species distributions.

File Name: Supplementary Data 2
Description: List of species occurring in checklists used in this study with species name (according to BirdLife International taxonomy), number of observations (in the checklists we used), forest-dependency, endemism to the hotspot, and red list status as used to build assemblage indices.
